# Mathematical Modeling of Sustainable Synaptogenesis by Repetitive Stimuli Suggests Signaling Mechanisms *In Vivo*

**DOI:** 10.1371/journal.pone.0051000

**Published:** 2012-12-20

**Authors:** Hiromu Takizawa, Noriko Hiroi, Akira Funahashi

**Affiliations:** Dept. of Bioscience and Informatics, Keio University, Yokohama, Japan; Georgia State University, United States of America

## Abstract

The mechanisms of long-term synaptic maintenance are a key component to understanding the mechanism of long-term memory. From biological experiments, a hypothesis arose that repetitive stimuli with appropriate intervals are essential to maintain new synapses for periods of longer than a few days. We successfully reproduce the time-course of relative numbers of synapses with our mathematical model in the same conditions as biological experiments, which used Adenosine-3′, 5′-cyclic monophosphorothioate, Sp-isomer (Sp-cAMPS) as external stimuli. We also reproduce synaptic maintenance responsiveness to intervals of Sp-cAMPS treatment accompanied by PKA activation. The model suggests a possible mechanism of sustainable synaptogenesis which consists of two steps. First, the signal transduction from an external stimulus triggers the synthesis of a new signaling protein. Second, the new signaling protein is required for the next signal transduction with the same stimuli. As a result, the network component is modified from the first network, and a different signal is transferred which triggers the synthesis of another new signaling molecule. We refer to this hypothetical mechanism as network succession. We build our model on the basis of two hypotheses: (1) a multi-step network succession induces downregulation of SSH and COFILIN gene expression, which triggers the production of stable F-actin; (2) the formation of a complex of stable F-actin with Drebrin at PSD is the critical mechanism to achieve long-term synaptic maintenance. Our simulation shows that a three-step network succession is sufficient to reproduce sustainable synapses for a period longer than 14 days. When we change the network structure to a single step network, the model fails to follow the exact condition of repetitive signals to reproduce a sufficient number of synapses. Another advantage of the three-step network succession is that this system indicates a greater tolerance of parameter changes than the single step network.

## Introduction

Synaptic plasticity is the physiological basis of learning and memory storage [1]–[3]. Long-Term Potentiation (LTP) is a type of synaptic plasticity and is thought to be the fundamental mechanism for the formation of memory. LTP consists of two distinguishable phases: the Early Phase of LTP (E-LTP) and the Late Phase of LTP (L-LTP). L-LTP is thought to contribute to long-term memory formation. L-LTP requires gene expression and protein synthesis and is accompanied by synaptic reorganization including synaptogenesis, the disappearance of synapses, and structural changes in synapses [4]–[10]. These structural changes to form memories and to establish learning are recognized to be equivalent to the various types of molecular signaling behavior [11]. Molecular level mechanisms of LTP have been elucidated, and recently many mathematical models based on these findings have been built and analyzed.

For example, some models are built focusing on CaMKII regulation as a prominent candidate for a bistable molecular switch, which induces L-LTP [12]–[16]. Other models, which include comprehensive knowledge of the LTP mechanism, also show bistable characteristics [17], or explain the synaptic pattern selectivity according to the intervals of external stimuli [18].

The mathematical models mentioned above focus on understanding the mechanism of induction or maintenance of LTP at a molecular level. They are efficient for representing and analyzing the dynamics of the molecules involved in LTP. One of the reasons why these models are suitable for the analysis of LTP induction and maintenance is that their timescale fits the phenomenon. However, the timescale of long-term memory is generally much longer than LTP, which occurs after the reorganization of cytoskeletal proteins in a spine. For example, in the experiments by Tominaga and Yamamoto [19]–[21], the newly produced synapses were maintained for 2 weeks. This is over 2,000,000 times longer than Ca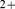 induction in the cytoplasm. Even compared with L-LTP, long-term maintenance of synapses requires 300 times longer. Therefore, we tuned the timescale of our model to be appropriate to represent long-term synaptic maintenance.

Tominaga and Yamamoto observed induction and maintenance of synapses for over 2 weeks by stimulating 3 or more times with appropriate time intervals (3 to 24 hrs). However, one or two stimuli, and even 3 stimuli within too short or too long a time interval, could not induce long-term maintenance of synapses [19]–[21]. Models of long-term synaptic maintenance should be distinct from the other models, not only because of the timescale but also because of the specific requirement to induce the phenomenon.

Therefore, we attempted to build a specific mathematical model, which focuses on synaptogenesis and long-term synaptic maintenance instead of shorter timescale models which focus on molecular events. The objective of building this model is to clarify the mechanism of synaptogenesis and long-term synaptic maintenance, which is expected to be the basic mechanism of memory formation and reinforcement in the newborn to mature brain [22]–[26]. Henceforth, we refer to our mathematical model as the sustainable synapse model.

Treatment with an inhibitor of protein synthesis, anisomycin, prevents L-LTP and subsequent long-term synaptic maintenance [8]. This fact led us to speculate that newly synthesized proteins play critical roles in synaptogenesis and the subsequent long-term synaptic maintenance.

**Figure 1:**
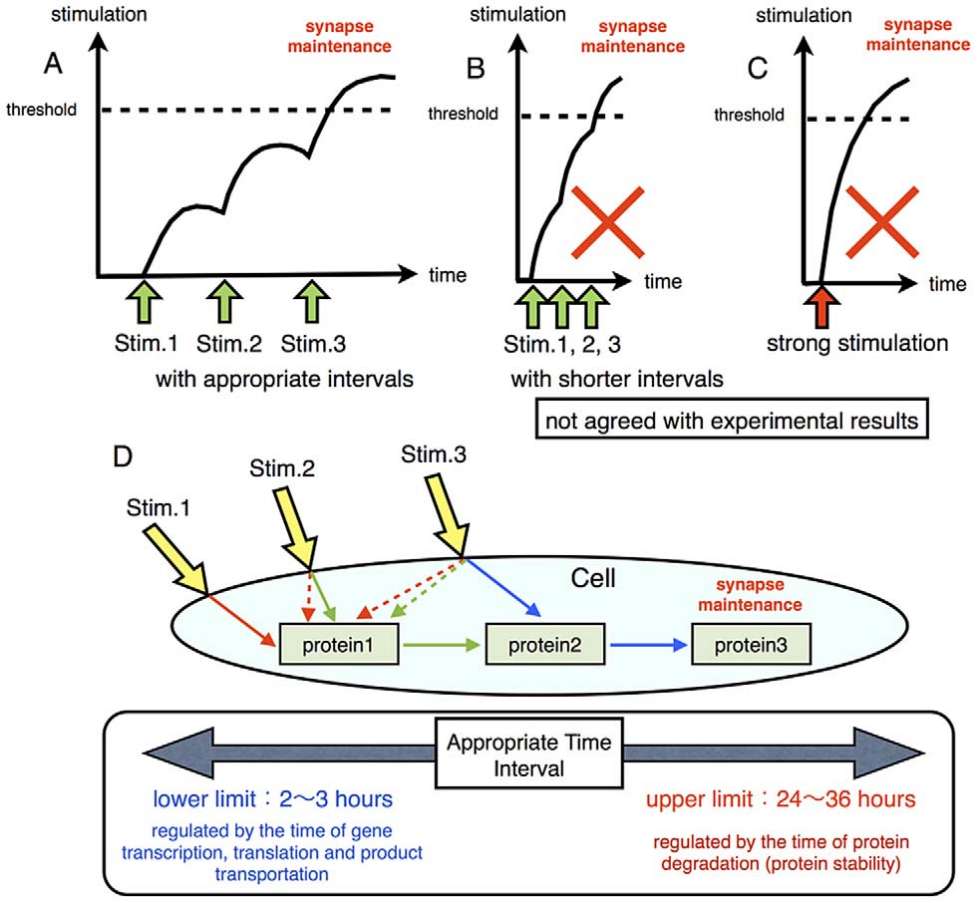
Possible mechanism for synaptic maintenance. A, B and C: synaptic maintenance occurs when stimulation exceeds the threshold. Repetitive stimuli with very short intervals (B) or a single strong stimulation (C) may exceed the threshold and induce synaptic maintenance. This mechanism cannot explain the necessity for repetitive stimuli with appropriate intervals (A). D: Repetitive stimuli with appropriate intervals induce different signal transduction cascades in each stimuli. At first, the first stimulation (Stim. 1) induces Protein 1 synthesis. Next, the second stimulation (Stim. 2) induces Protein 2 synthesis via Protein 1. Lastly, the third stimulation (Stim. 3) induces Protein 3 synthesis with the intermediation of Protein 2. Protein 3 may play a direct and important role in long-term synaptic maintenance.

If overcoming a threshold of the amount of the synthesized protein regulates long-term synaptic maintenance, both repetitive stimuli with shorter intervals and a single strong stimulation would be sufficient to induce long-term synaptic maintenance (Figure 1**A** to **C**). However, experimental results exclude these possibilities [19]–[21].

In contrast, our hypothetical mechanism of network succession can explain why repetitive stimuli with appropriate time intervals are required for long-term synaptic maintenance (Figure 1**D**). In this mechanism, the signaling molecule for the next step is produced by the previous stimulation. As a result, when the cell receives the next external stimulus, the signaling network is transformed into a different set of molecules, including the protein newly produced by the previous stimulus. This newly formed signaling network induces the next signaling molecule, which is different from the molecule required at the second step. This orderly processes of change in signal transduction brought about by the progressive replacement of one protein by another will keep going until a stable climax is established, in this case the long-term maintenance of synapses.

This hypothesis basically requires newly synthesized proteins for the succession of the signaling network. The requirement of the new generation of signaling molecules guarantees that the mechanism needs a longer interval than the time for protein synthesis. This means that the time needed for protein synthesis determines the minimum duration of interval between external signals. The commonly known time to generate active proteins from genes via transcription and translation following their transportation and modification is a few hours. This is compatible with the shortest duration of intervals between external stimuli in experiments [19]–[21]. Protein modification such as phosphorylation takes a much shorter time, as we can see in the example of the MAPK cascade [27], [28]. We estimate that protein modification may be involved in but not an essential part of the reconstruction of the experimentally observed phenomena.

At the same time, because network succession requires the existence of the generated protein, the upper limit of the intervals between external signals depends on the stability or lifetime of these newly synthesized proteins. Many signaling proteins are degraded within 48 hrs [29]. This fact is also in keeping with the experimentally observed phenomena [19]–[21].

The number of steps of the network succession is effective in the whole dynamics of the system. We assume that the mechanism for long-term synaptic maintenance consists of a three-step succession of signaling networks. One of the reasons for this assumption is the result of the experiments of Tominaga and Yamamoto [19]–[21] which indicated that stimuli repeated more than three times are required for long-term synaptic maintenance.

It is also suggested from the experimental results that the condition of the cell is altered by repetitive stimuli. Kawaai reported that stimuli repeated at 24 hr intervals altered gene expression patterns [30]. In their research, stimuli repeated twice increased gene expressions of PAK4, LIMK, SSH, COFILIN and YWHAZ, and promoted the reorganization and turnover of actin filament (Figure 7.B in [30]). Because actin dynamics directly relates to synaptic reorganization [31]–[33], the reorganization and turnover of actin filament may cause a transient increase and degradation of unstable synapses. However, after the third stimulation, the expression levels of PAK4, LIMK and YWHAZ were increased in the same manner as those in the first and the second stimulations, but the expression levels of COFILIN and SSH were decreased [30]. Because of the effect of COFILIN and SSH on F-actin [34]–[36], a decrease in these molecules leads to stabilization of F-actin following long-term synaptic maintenance. We assume that decreasing the expression level of COFILIN and SSH was directly connected to the induction of stable synapses. Based on the experimental information, we built a three-step network succession model, and compared the dynamics with the modified model which consists of a single step network, especially with regard to the sensitivity to changes in the parameters.

At the same time, we adopted this mechanism as an actin reorganization/stabilization mechanism in our sustainable synapse model.

**Figure 2:**
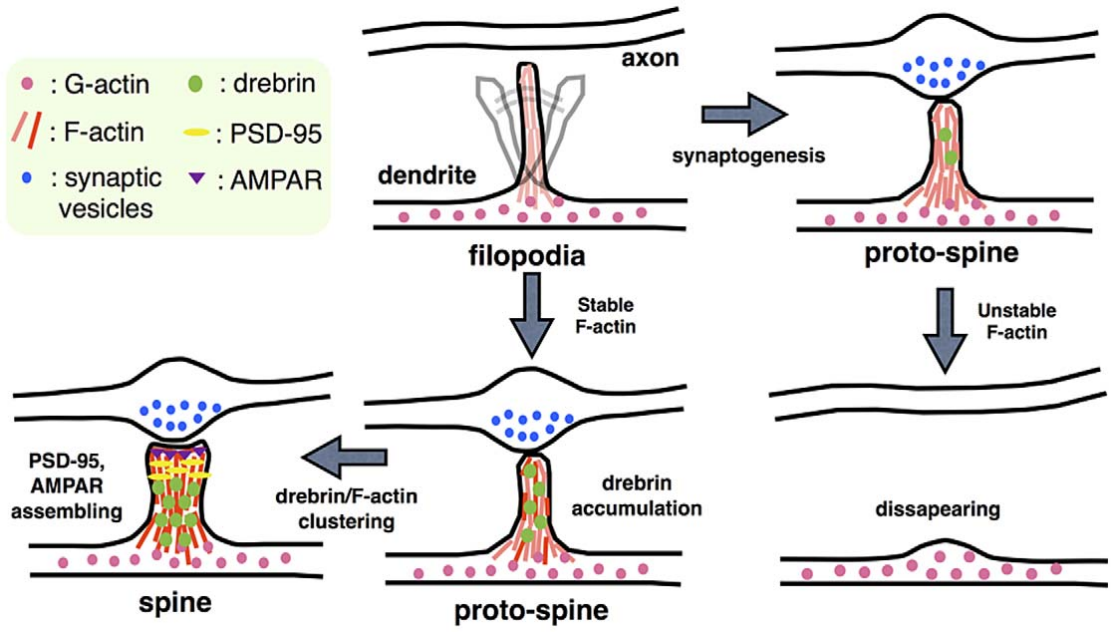
Synapse stabling mechanism. Drebrin induces the assembly of PSD-95 and AMPAR in dendritic protrusions through F-actin accumulation and stabilization, and causes synaptic maintenance. The color difference of F-actin indicates whether the fiber is stable (red) or unstable (pink).

We used other biological knowledge to determine how to define the basic structure of our model. This relates to the formation of dendritic protrusions, such as filopodia and spines, which are significant for synaptogenesis and synaptic maintenance. A summary of the mechanism is given in Figure 2. The first step of synaptogenesis is contact between the neuronal axon and dendritic filopodia. The filopodia are the precursors of spines. The processes of transition from filopodia to spine have been investigated [37]–[40]. The dendritic filopodia grow into protospines, which are an intermediate form between the filopodia and spines. The stability, shape and motility of these dendritic protrusions are related to the dynamics of the F-actin [40]–[43]. In particular, dynamic F-actin, which is actively polymerized, depolymerized and turned over, is reorganized in the filopodia and protospines. These dynamics lead to the disappearance and regeneration of synapses. The dynamics of F-actin are controlled by various actin binding proteins such as Arp2/3, Cortactin, Profilin, and ADF/COFILIN. Genetic modifications of these proteins affect the structure, the number and the density of spines and synapses [44].

Takahashi et al. found that Drebrin, one of the actin binding proteins, plays a key role in stabilizing protospines [45]. They suggested that the stabilization of protospines requires an accumulation of Drebrin and successful clustering of Drebrin with skeletal F-actin of the protospines. Because Drebrin inhibits actin-myosin interaction, F-actin clustered with Drebrin is further stabilized and this leads to stable accumulation of PSD-95, which is one of the main components of post synaptic density (PSD). PSD is the characteristic structure in the tip of the head of a spine [45]. Then, PSD anchors synaptic receptors [46]–[48] and the anchored receptors induce activity dependent enlargement of the spine head [49]. Finally, the enlarged spine head is stiffly connected to the presynaptic active zone by cell adhesion molecules [50]. After establishing the broad contact with axon, a large number of excitatory synapses are formed on the dendritic spines [51]. The newly formed synapses are stabilized by the sequential processes in the above. The first critical step for stabilizing protospines is the accumulation of Drebrin following the clustering of Drebrin with F-actin. Therefore, we defined F-actin-Drebrin clustering as the trigger for the stabilization of mature synapses in our model.

Thus, our sustainable synapse model stands on the two hypotheses that: 1) a multi-step network succession induces the downregulation of SSH and COFILIN gene expression which triggers the production of stable F-actin; 2) the formation of the complex of stable F-actin with Drebrin in PSD is the critical mechanism to produce long-term synaptic maintenance. A summary of the modeled mechanism of long-term synaptic maintenance is as follows: repetitive stimuli with appropriate time intervals decrease SSH and COFILIN. The decrease in these molecules induces transient stabilization of F-actin. Drebrin starts to cluster with transiently stabilized F-actin for further stabilization. Finally, the protospine grows into a mature synapse with appropriate receptors on the plasma membrane.

**Figure 3:**
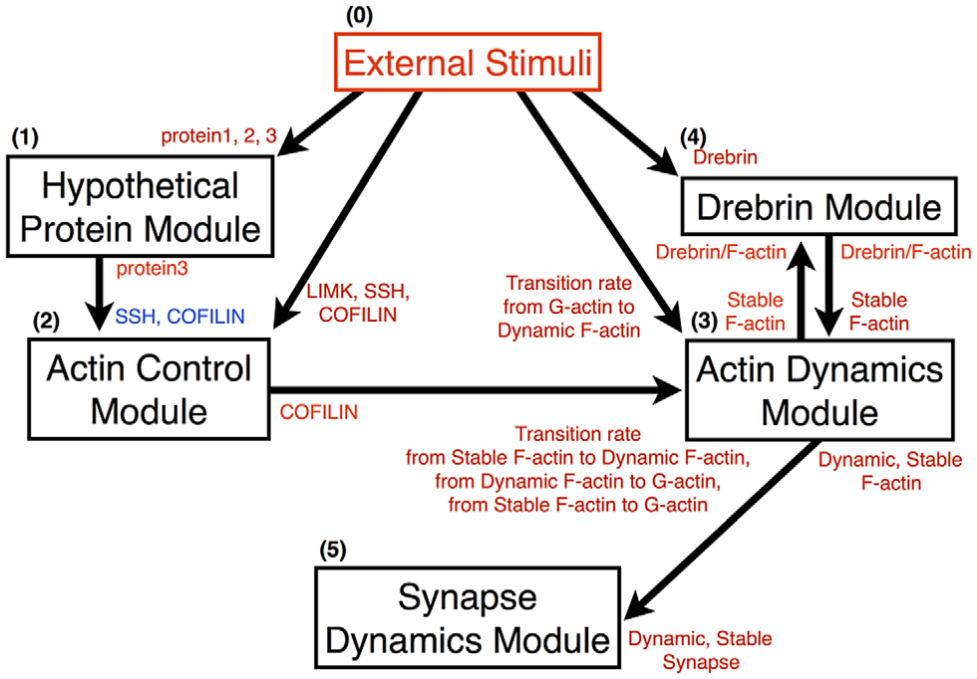
Interactions of each module. The arrows indicate the directions of effects between two modules. The components of each module shown beside the shafts of arrows affect the other components shown beside the arrowheads. The red/blue symbols indicate an increase/decrease in components in the simulation.

**Figure 4:**
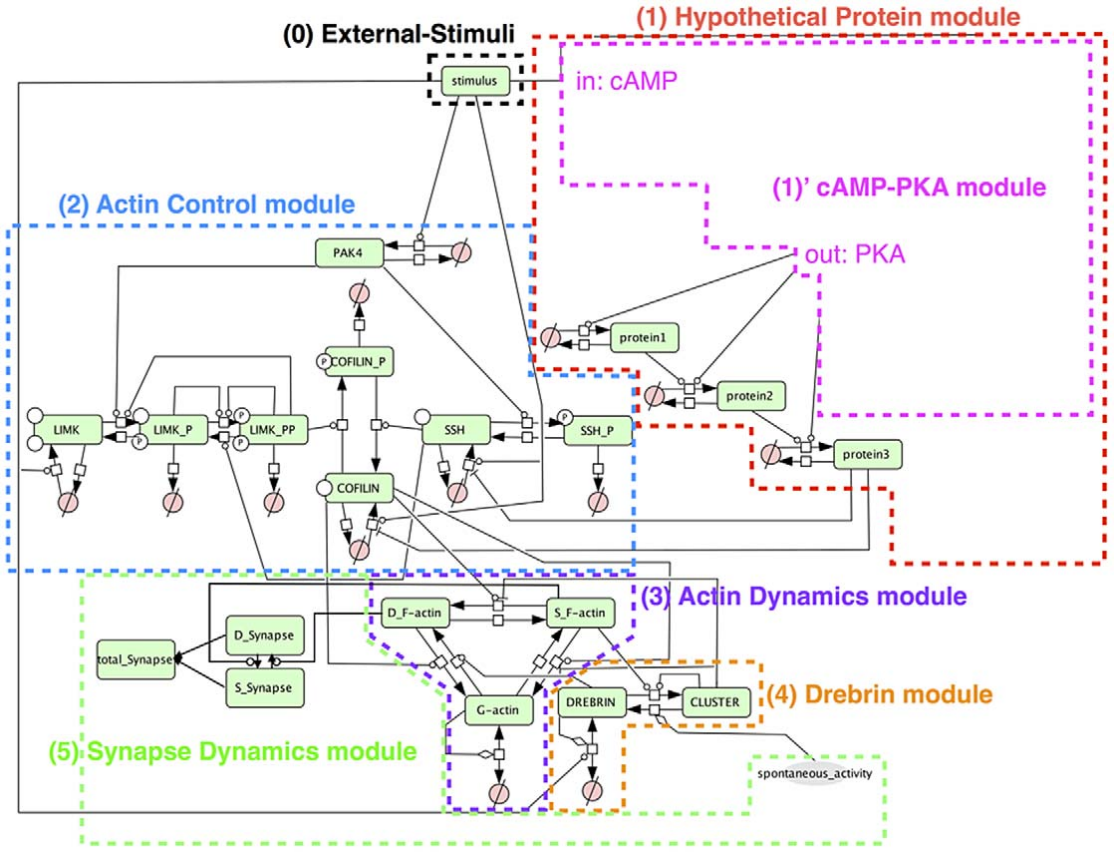
Whole view of the model. The model is written in accordance with Systems Biology Graphical Notation (SBGN) [83]. 0. Black box: External stimulation exists at the most upstream and drives our model. 1. Red box: Hypothetical protein module includes hypothetical proteins which play an important role in possible network succession for long-term synaptic maintenance. 2. Blue box: Actin control module includes actin binding protein: COFILIN and COFILIN kinase/phosphatase. 3. Purple box: Actin dynamics module includes G-actin and F-actin to explain the actin dynamics (polymerization and depolymerization). 4. Orange box: Drebrin module includes the actin binding protein: Drebrin. Drebrin affects actin dynamics and is clustered with F-actin. 5. Green box: Synapse dynamics module includes two kinds of synapses to explain the dynamics of synaptogenesis and synaptic maintenance. The white square boxes linked to two connectors are process nodes, which represent processes that transform one or several entity pools to be identical or different. The circles crossed by a bar linking the upper-right and lower-left corners of an invisible square drawn around the circle (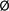) are empty sets which represent the source or sink [84].

**Figure 5:**
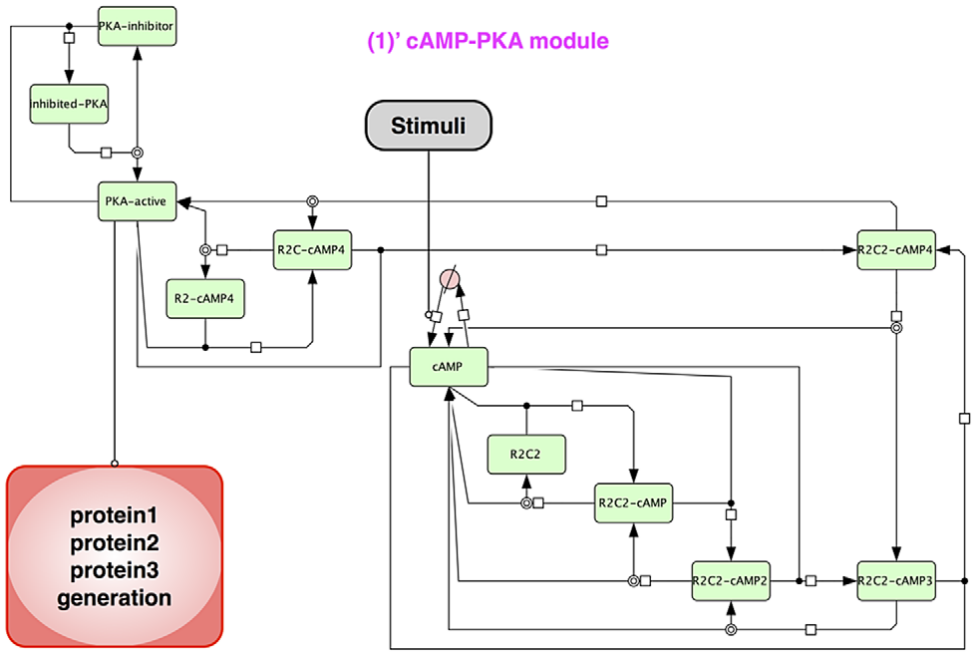
Structure of cAMP-PKA module. This module expresses the activation mechanism of PKA with cAMP. The PKA complex (R2C2) consists of two catalytic units and regulatory (inhibitory) units. External stimuli increase cAMP and four cAMP bind to R2C2. The R2C2-cAMP4 complex releases two catalytic units (PKA-active).

The relationship between each module is indicated in Figure 3, and the entire model is shown in Figure 4. Also the cAMP-PKA module is shown separately in Figure 5.

## Materials and Methods

### Mathematical model descriptions

Our mathematical model is composed of five modules and each module is created on the basis of the following three biological facts and the hypotheses derived from them. 1: A transition in actin dynamics from a dynamic state to a stable state with alteration in the gene expression pattern. 2: A transition mechanism from filopodia to spine (changes of the F-actin stability in the dendritic protrusion). 3: Hypothetical network succession for the alteration in the gene expression pattern. We show the details of each module below. Each module is explained in the following subsection.

Our basic sustainable synapse model includes a three-step network succession.

The source code of our sustainable synapse model (an SBML file) and the solver library which is required to simulate our model are being made available on our laboratory's webpage (http://www.fun.bio.keio.ac.jp/softwares) for free download.

#### External stimulation

**Figure 6:**
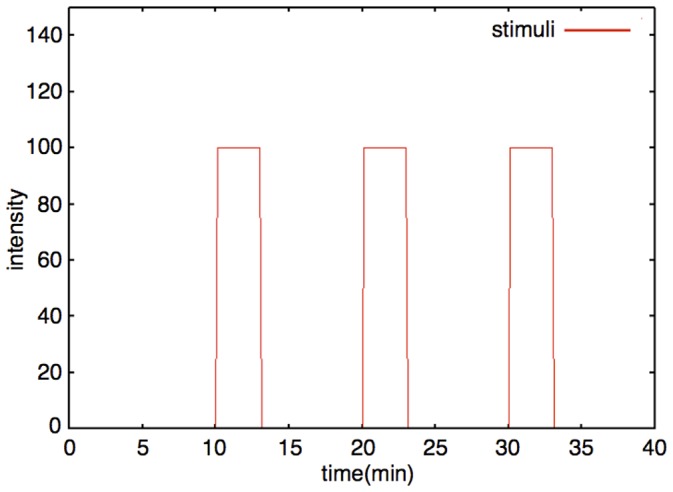
Example of external stimuli. The red line is an example of the dynamics of external stimuli (Number of stimuli: 3; Interval: 10 min; Duration time: 3 min; Intensity: 100).

**Figure 7:**
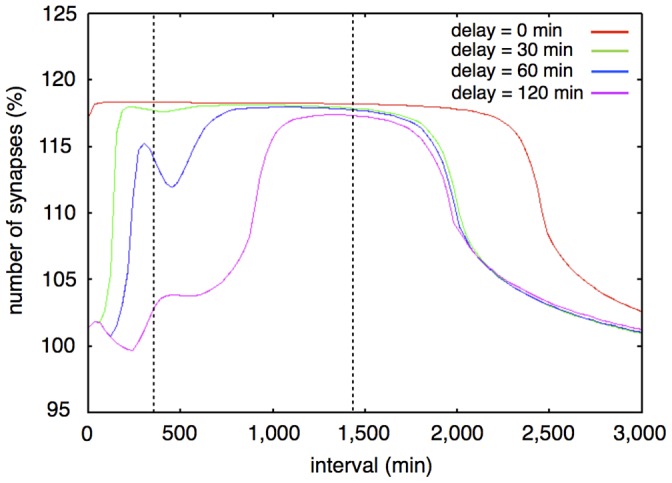
Synaptic maintenance responsiveness of our model to intervals (three times repetitive stimuli, with various lengths of delay). The horizontal axis shows the intervals of each stimulation. The vertical axis shows the ratio of increase of synapses at two weeks after the first stimulation. The colored lines show the synaptic maintenance response with various *delays*. Red line: 0 min (no delay); Green line: 30 min; Blue line: 60 min; Purple line: 120 min. The two vertical dotted lines show the approximate upper limit and lower limit of intervals between three times stimuli for synaptic maintenance in experimental results [20].

External stimulation exists most upstream in our model and works as the motive force for the stimuli responsive model system. We supposed the stimuli to be Sp-cAMPS in our model as the inducer of LTP. We used a rectangular wave to explain these stimuli. We can control following properties of stimuli. 1: The number of stimuli. 2: The length of intervals between stimuli. 3: The duration of stimuli. 4: The intensity of the stimuli. A typical external stimuli is shown in Figure 6. The response of this model to the various intervals of external stimuli is indicated in Figure 7.

#### Hypothetical protein module

The Hypothetical protein module includes a cAMP-PKA module, hypothetical proteins 1, 2, and 3. This module is completely based on the hypotheses postulated on the basis of the experimental results [19]–[21], with the exception of the upstream cAMP-PKA module.

**Figure 8:**
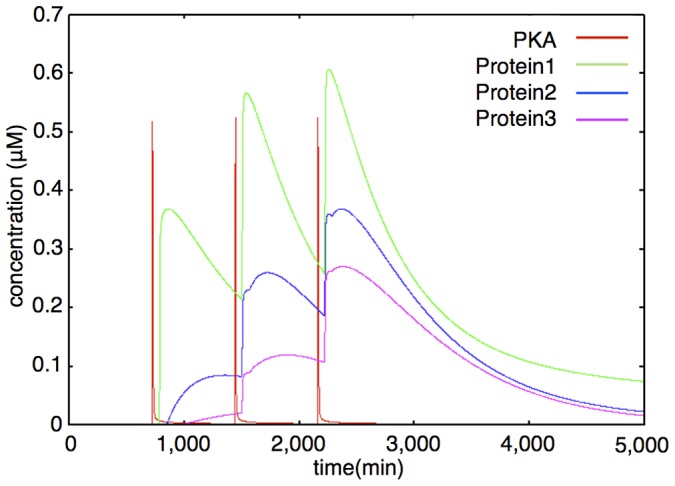
Example of dynamics of PKA, Proteins 1, 2, 3 (interval between stimuli = 720 min). The colored lines show the time-course fluctuations of each of the components. Red: PKA; Green: Protein 1; Blue: Protein 2; Purple: Protein 3. External stimuli increase cAMP level (not shown) and cAMP increases the PKA level. PKA directly increases Protein 1 level. Protein 1 and PKA coordinately increase Protein 2. Protein 2 and PKA coordinately increase Protein 3. Protein generation is time-consuming, so there are time-lags between the PKA increase and protein increase.

To express the required time of gene expression and protein synthesis, each rate equation includes a delay (which appears as the parameter, *delay*). An example of the dynamics of the Hypothetical protein module is shown in Figure 8. A time-course simulation showed that this module worked to implement the processes of network succession in this signaling network. The first stimulation increases Protein 1 (Figure 8, green), the second stimulation increases Protein 2 (Figure 8, blue) via Protein 1, and the third stimulation increases Protein 3 (Figure 8, purple) via Protein 2. Rate equations of the Hypothetical protein module are shown in the following subsection (Rate Equations of the Mathematical Model).

The structure and the parameters of the cAMP-PKA module follow the comprehensive mathematical model, which was originally built for Bhalla's research [52] and was refined in Ajay's research ([18], Figure 3**B** in the reference). Briefly, this module consists of various states of cAMP and PKA complex, and also the states of the PKA catalytic domain with its inhibitor. PKA is formed by a holoenzyme, in which catalytic (C) and regulatory (R) subunits are associated. The C subunit contains the active center, whereas the R subunit has two cAMP binding sites. This C and R subunit complex is in an inactive state. The binding of cAMP to the R subunit induces C subunit activation, accompanied by the dissociation of the R subunit from the C subunit [53]. There exists a physiological PKA inhibitor, such as a heat-stable protein kinase inhibitor (PKI). PKI is one of the key players in the regulation of activity and in localizing the C subunit [54]. PKI is also involved in neuronal signal transduction with effects on learning and memory by affecting LTP [55]. The signal of PKA activation is reflected in the expression of the hypothetical Proteins 1 to 3.

#### Actin control module

**Figure 9:**
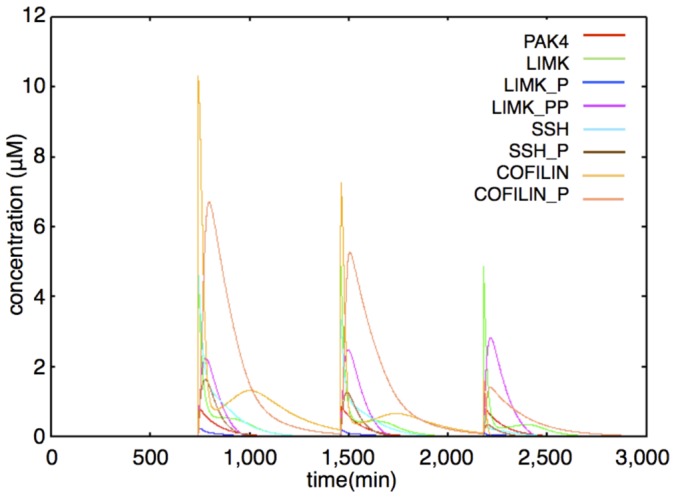
Example of dynamics of PAK4, LIMK, SSH, COFILIN (interval between stimuli = 720 min). Red: PAK4; Green: LIMK; Blue: LIMK P; Purple: LIMK PP; Light blue: SSH; Brown: SSH P; Amber: COFILIN; Pink: COFILIN P. PAK4, LIMK, SSH and COFILIN are increased by external stimuli. Immediately after stimuli, LIMK decreases and LIMK PP increases by PAK4 and self-phosphorylation, SSH decreases and SSH P increases by PAK4 and then COFILIN decreases and COFILIN P increases by LIMK PP. Because the generation velocities of SSH and COFILIN are decreased by repetitive stimuli, the COFILIN level becomes very low after the third stimulation.

We built this module according to Breindl's model [56]. The Actin control module includes PAK4, LIMK, SSH and COFILIN. PAK4 and the Non-phosphorylated state LIMK, SSH and COFILIN are generated by external stimulation. LIMK and SSH are phosphorylated into LIMK_P and SSH_P by PAK4. LIMK has two phosphorylated states, the single phosphorylated state (LIMK_P) and the double phosphorylated state (LIMK_PP). LIMK_P is phosphorylated into LIMK_PP by auto-phosphorylation. SSH and COFILIN have only a single phosphorylated state (SSH_P, COFILIN_P). COFILIN is phosphorylated by LIMK_PP and COFILIN_P is dephosphorylated by SSH. Most of the reactions in the Actin control module are phosphorylation and dephosphorylation. Therefore, we describe the rate equations as Michaelis-Menten kinetics, except for production and degradation. Production of PAK4, LIMK, SSH and COFILIN has a delay after the external stimulation because of the time of gene expressions and protein synthesis. An example of the dynamics of the Actin control module is shown in Figure 9. The time-course simulation in this module succeeded in reproducing the changes in the synthesis pattern of the actin-related proteins, which are caused by the changes in the gene expression pattern with repetitive stimuli [30]. Rate equations of the Actin control module are shown in the following subsection (Rate Equations of the Mathematical Model).

#### Actin dynamics module

**Figure 10:**
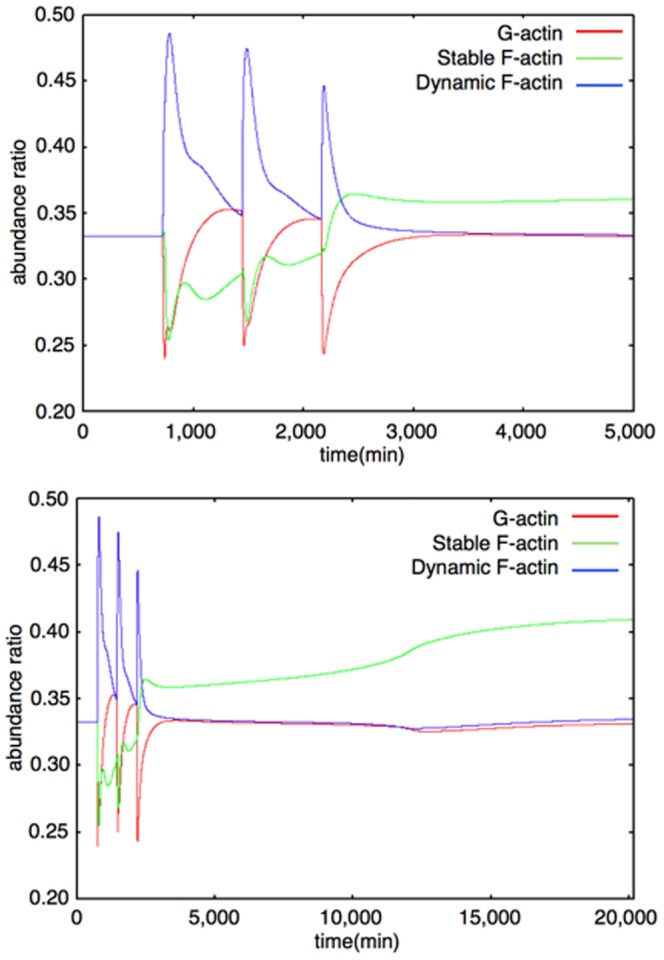
Example of dynamics of G-actin, Dynamic F-actin, Stable F-actin (interval between stimuli = 720 min). Upper Figure: 0–5000 min, Lower Figure: 0–20160 min (14 days) The colored lines show time-course fluctuations of each of the components. Red: G-actin; Green: Stable F-actin; Blue: Dynamic F-actin. External stimuli directly decrease G-actin and increase Dynamic F-actin. Stable F-actin is decreased by the effect of COFILIN. After the third stimulation, the levels of G-actin and Dynamic F-actin change in almost the same manner as before, but Stable F-actin hardly decreases because the COFILIN level is very low and the Drebrin/F-actin cluster stabilizes F-actin at this time and the Drebrin/F-actin cluster increases the Stable F-actin level in a self-feedback manner.

**Figure 11:**
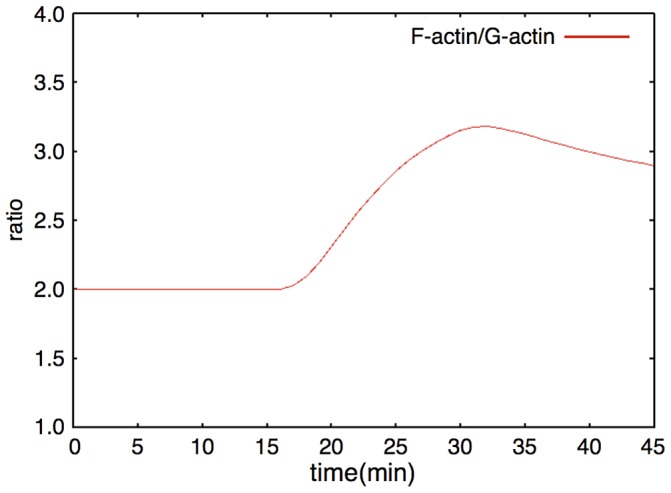
F-actin/G-actin ratio. The red line shows the time-course fluctuations of the F-actin/G-actin ratio. F-actin includes Dynamic and Stable F-actin. A single stimulation induces an increase in the F-actin/G-actin ratio.

We represent the actin dynamics in this module. Actin has two main states: G-actin (monomer state) and F-actin (polymer, filament state). We also divide F-actin into two groups: Dynamic F-actin and Stable F-actin, based on its stability. Dynamic F-actin represents the F-actin in the state of turnover or very active reorganization. Stable F-actin represents the F-actin which seldom changes its structure and is maintained over a long period of time. Each of the states of actin (G-actin, Dynamic and Stable F-actin) are able to transfer their states to each other. F-actin stability is defined by the actin turnover ratio, which is measured by FRAP analysis [31]. An example of the dynamics of the Actin dynamics module is shown in Figure 10. The time-course simulation in this module succeeded in reproducing the characteristic phenomena that LTP inducing stimuli increase the F-actin content in spines [32], [33]. We were also able to reproduce the F-actin/G-actin ratio increasing phenomenon in a mathematical model simulation (Figure 11). Rate equations of the Actin dynamics module are shown in the following subsection (Rate Equations of the Mathematical Model).

#### Drebrin module

**Figure 12:**
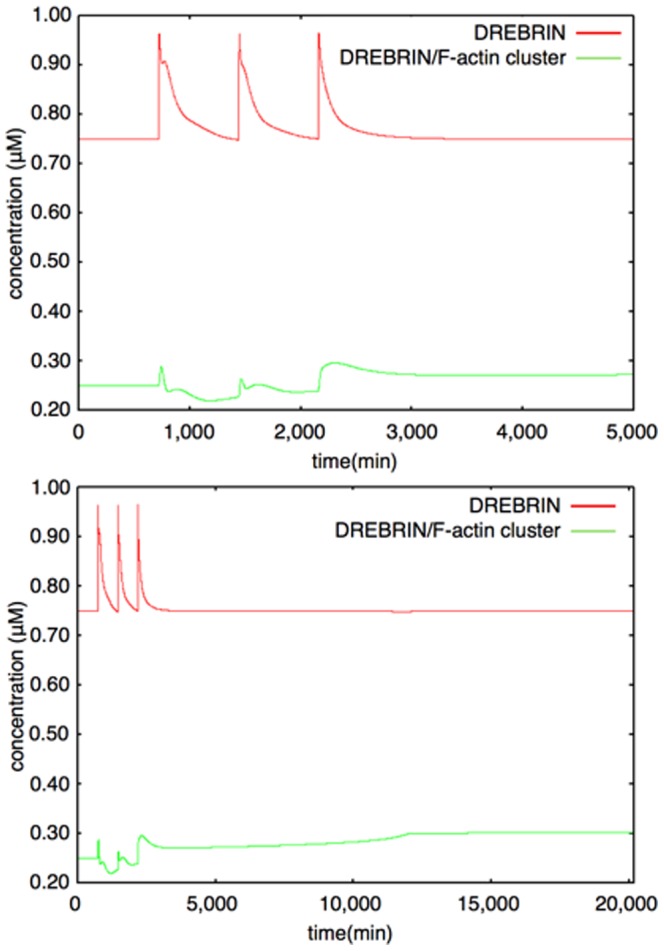
Example of dynamics of Drebrin (interval between stimuli = 720 min). Upper Figure: 0–5000 min, Lower Figure: 0–20160 min (14 days) The colored lines show the time-course fluctuations of each of the components. Red: Drebrin; Green: Drebrin/F-actin cluster. External stimuli increase the Drebrin level. The Drebrin/F-actin cluster increases dependence on Drebrin and the Stable F-actin level. The equilibrium value of the Drebrin/F-actin cluster rises after the third stimulation because the Stable F-actin level increases at that time and once a large cluster is generated, a high equilibrium value is maintained in a self-feedback manner.

To build this Drebrin module, we considered two states of Drebrin: the dispersed state (Drebrin) and the clustered state (Drebrin/F-actin complex cluster). The dispersed state shows the state of Drebrin which exists in spines sparsely and does not form a cluster. The clustered state shows the state of Drebrin which exists densely in spines as a cluster with F-actin. A rate equation of cluster formations is expressed by using the Hill equation [57], which is often used to express orchestrated binding. A Drebrin/F-actin cluster causes further clustering by stabilizing the F-actin skeleton. We also considered Drebrin accumulations in dendritic protrusions, such as filopodia, protospine and spine, because Drebrin accumulates in them, when L-LTP is induced [19], [20]. An accurate mechanism of Drebrin accumulations has not yet been clarified, so we assumed that external stimuli simply increase Drebrin in our mathematical model. However, synapses are also maintained by spontaneous activity of the neuron [20], [58]. There may be a mechanism whereby extra synapses are trimmed if synapses increase to a larger extent than the limited amount which is maintained by spontaneous activity. In fact, there are experimental results that show that the number of synapses decreases when spontaneous neuronal activity is blocked by Tetrodotoxin (TTX) [20]. An example of the dynamics of a Drebrin module is shown in Figure 12. The time-course simulation in this module succeeded in reproducing the phenomena that accumulations of Drebrin in spines and the clustering of Drebrin with F-actin are produced by LTP inducing stimuli. Rate equations of the Drebrin module are shown in the following subsection (Rate Equations of the Mathematical Model).

#### Synapse dynamics module

**Figure 13:**
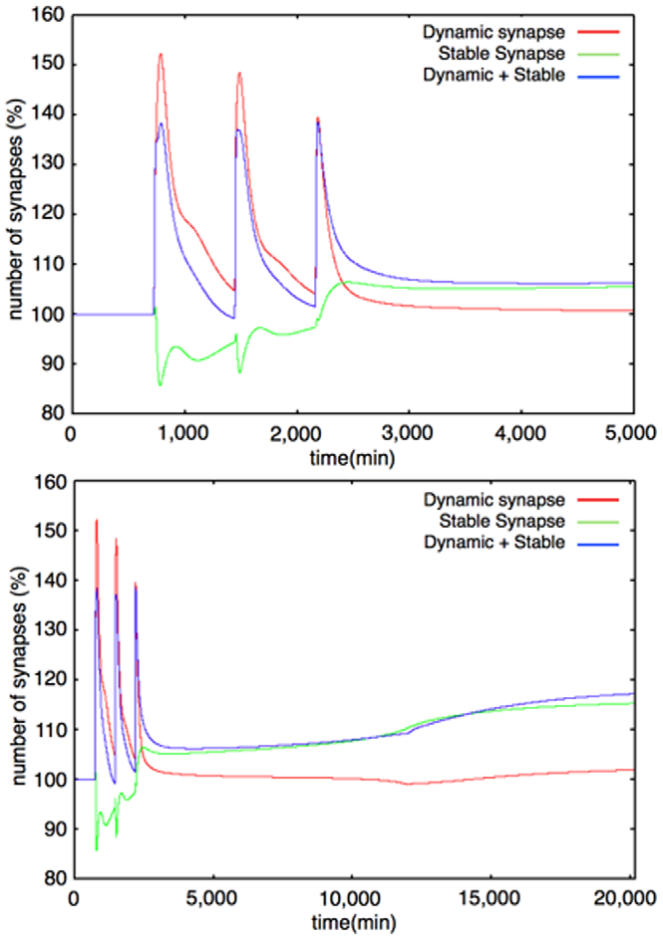
Example of dynamics of synapses (interval between stimuli = 720 min). Upper Figure: 0–5000 min, Lower Figure: 0–20160 min (14 days) The colored lines show the time-course fluctuations of each of the components. Red: Dynamic Synapse; Green: Stable Synapse; Blue: Total Synapse (Dynamic+Stable). The Dynamic/Stable Synapse levels change according to the Dynamic/Stable F-actin levels. The time-course fluctuations of the synapses are similar to those of the Actin dynamics module. All the values in the graphs are normalized with the initial value of each variable, and indicate the relative values compared with the initial condition.

In the Synapse dynamics module, we considered two states of synapses. One is “Dynamic Synapses”, which has Dynamic F-actin as the post-synaptic spine skeleton and actively reorganizes its structure. The other is “Stable Synapses”, which has Stable F-actin as the post-synaptic spine skeleton and remains for a long time as it is. Dynamic Synapses fluctuate in coordination with Dynamic F-actin, and Stable Synapses fluctuate in coordination with Stable F-actin. The number of Stable Synapses exceeds the number of Dynamic Synapses when a larger amount of Stable F-actin exists than in the equilibrium state. This case is considered to correspond with the process whereby actin polymerization occurs actively in the spine head to stabilize the spines. An example of the dynamics of the Synapse dynamics module is shown in Figure 13. The time-course simulation in this module succeeded in reproducing synaptogenesis and synaptic maintenance with repetitive stimuli. The rate equations of the Synapse dynamics module are shown in the following subsection (Rate Equations of the Mathematical Model).

### Rate Equations of the Mathematical Model

The following abbreviations are used in this subsection:

Dynamic F-actin→D_F-actin Stable F-actin→S_F-actin

Dynamic Synapse→D_Synapse Stable Synapse→S_Synapse

These three actin states, G-actin, Stable F-actin, and Dynamic F-actin express the relative concentrations of these species, respectively. *stimulation* means the non-unit strength of the external stimuli.

All the other variables express the concentration of species which appear in Figure 4 and Figure 5 with the same names. The values and units of parameters and the initial conditions are listed in Tables 1, 2, 3, 4, 5, 6.

**Table 1:** Parameters of the model: Hypothetical protein module.

name	value	unit
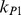	3240	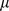 M 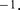 min 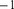
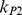	1980	min 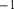
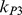	3240	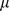 M 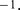 min 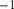
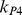	1980	min 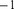
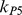	4500	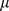 M 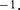 min 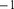
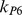	6600	min 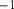
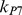	4500	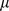 M 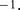 min 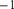
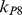	1950	min 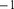
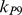	3600	min 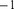
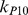	1080	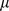 M 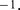 min 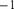
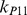	3600	min 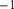
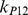	1080	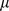 M 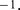 min 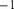
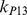	3600	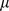 M 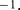 min 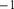
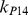	60	min 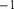
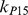	0.01	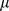 M  min 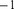
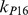	1.0	min 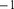
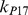	0.125 (0.0175*)	min 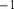
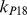	0.0025 (0.00175*)	min 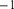
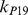	0.225	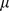 M 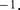 min 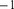
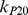	0.00175	min 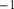
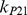	0.275	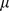 M 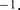 min 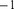
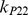	0.00175	min 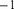

* single step model.

**Table 2:** Parameters of the model: Actin control module.

name	value	unit
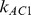	3.00E-3	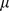 M  min 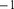
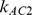	1.00E-2	min 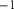
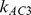	1.75E-2	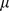 M  min 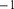
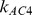	1.00E-2	min 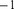
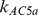	5.00E-1	min 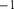
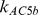	5.00	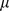 M
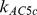	5.00E-3	min 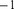
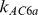	5.00E-1	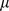 M  min 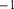
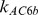	2.00	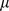 M
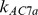	6.00E-1	min 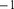
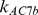	1.00E-1	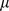 M
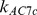	2.00	min 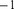
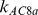	1.00E-1	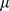 M  min 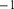
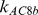	5.00E-1	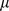 M
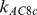	1.00E-1	min 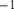
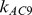	1.00E-2	min 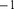
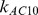	1.00E-2	min 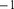
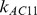	1.75E-2	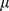 M  min 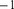
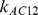	1.00E-2	min 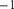
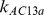	2.00E-1	min 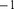
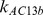	2.00	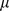 M
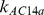	1.00E-1	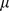 M  min 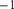
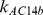	2.00	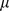 M
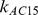	1.00E-2	min 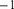
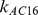	3.50E-2	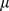 M  min 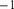
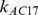	5.00E-3	min 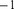
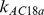	3.00E-1	min 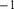
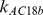	5.00	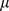 M
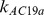	1.00E-1	min 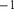
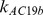	5.00	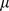 M
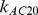	1.75E-1	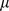 M 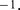 min 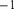
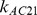	2.0E-3	min 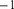
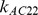	1.75E-1	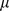 M 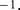 min 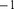
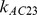	2.0E-3	min 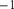

**Table 3:** Parameters of the model: Actin dynamics module.

name	value	unit
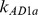	1.00E-1	min 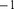
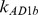	1.00E-1	–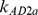	1.00E-3	min 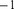
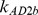	1.00E-1	–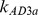	1.00E-1	min 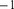
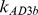	1.00E-1	–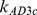	1.00E-4	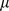 M 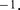 min 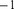
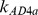	1.00E-2	min 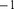
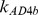	1.00E-1	–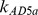	1.00E-3	min 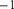
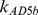	1.00E-1	–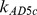	2.00E-4	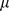 M 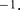 min 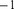
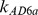	1.00E-2	min 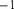
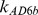	1.00E-1	–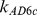	4.00E-4	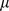 M 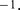 min 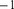
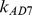	7.5E-2	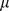 M 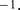 min 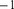
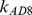	7.5E-1	min 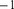
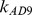	1.00E-3	min 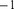
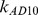	5.00E-6	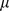 M 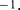 min 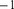
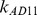	1.0E-1	min 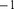
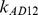	4.0E-4	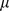 M 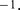 min 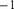
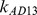	1.0E-3	min 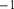

**Table 4:** Parameters of the model: Drebrin module.

name	value	unit
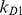	7.50E-4	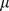 M  min 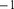
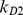	7.50E-3	min 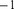
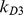	5.00E-2	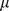 M 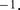 min 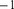
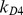	3.00E-1	–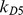	5.00E-2	min 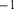
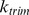	1.00E-2	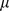 M  min 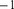
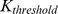	3.28E-1	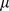 M
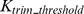	60.0	–

**Table 5:** Parameters of the model: Synapse dynamics module.

name	value	unit
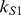	3.50	–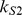	1.75E-1	–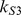	2.00	–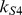	1.00E-1	–

**Table 6:** Initial values of the model species.

name	value	unit
LIMK	0.0	–
LIMK P	0.0	–
LIMK PP	0.0	–
COFILIN	0.0	–
COFILIN P	0.0	–
SSH	0.0	–
SSH P	0.0	–
PAK4	0.0	–
G-actin	0.333	–
S F-actin	0.333	–
D F-actin	0.333	–
stimulus	0.0	–
cAMP	0.0	–
protein1	0.0	–
protein2	0.0	–
protein3	0.0	–
DREBRIN	0.75	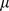 M
CLUSTER	0.25	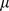 M
Spontaneous activity	78	–
D Synapse	0.0	–
S Synapse	0.0	–
Total Synapse	0.0	–
PKA-active	0.0	–
R2C2	0.0	–
R2C2-cAMP	0.0	–
R2C2-cAMP2	0.0	–
R2C2-cAMP3	0.0	–
R2C2-cAMP4	0.0	–
R2C-cAMP4	0.0	–
PKA-inhibitor	0.25	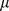 M
inhibited-PKA	0.0	–
R2-cAMP4	0.0	–

#### Rate equations of Hypothetical protein module



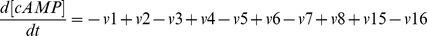


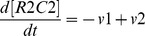


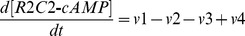


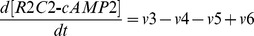


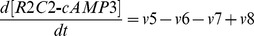


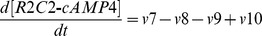


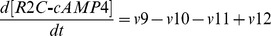


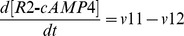


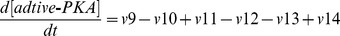


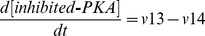


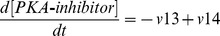


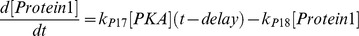


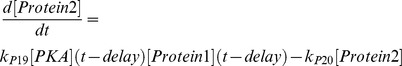


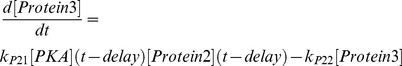


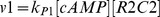


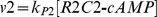


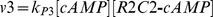


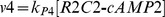


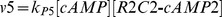


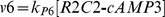


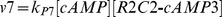


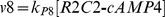


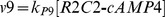


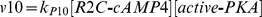


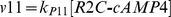


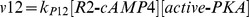


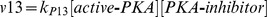


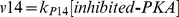


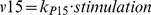


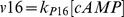



#### Rate equations of Actin control module



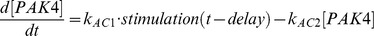


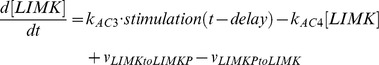


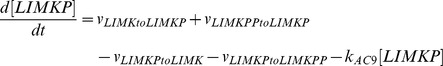


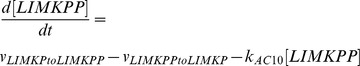


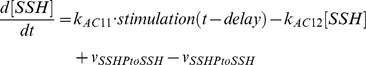


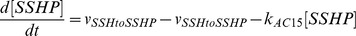


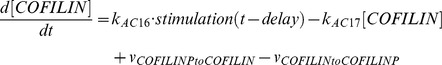


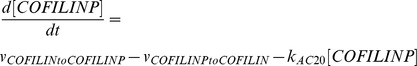


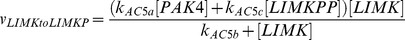


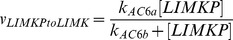


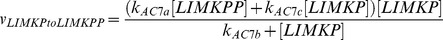


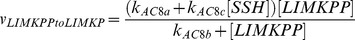


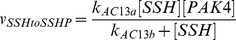


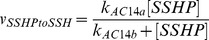


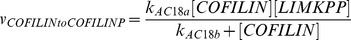


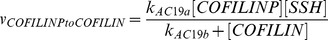


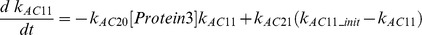


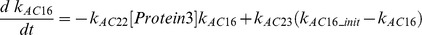



#### Rate equations of Actin dynamics module



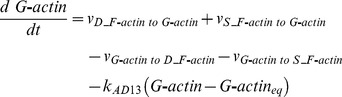


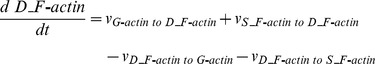


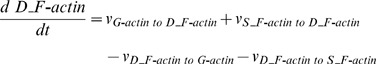


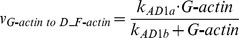


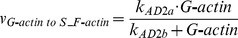


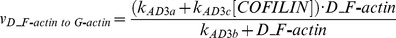


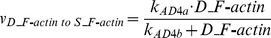


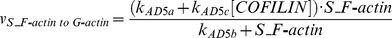


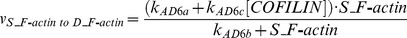


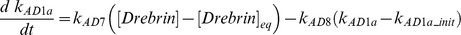


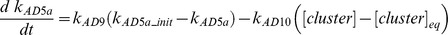


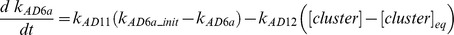



#### Rate equations of Drebrin module



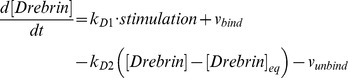


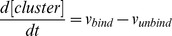


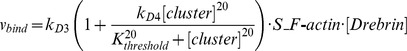


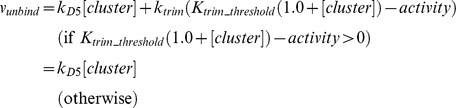



#### Rate Equations of Synapse dynamics module



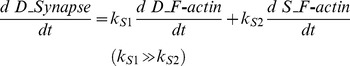


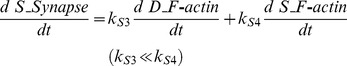



### Simulation Methods

Our mathematical model is written in SBML [59] Level 2 Version 4. It is possible to alter or extend its structure and equations easily with SBML supporting software such as CellDesigner [60]. Because there are few simulators which support Delayed Differential Equations (DDEs) with SBML, we built our own simulator, originally in C using libSBML [61] and the basic C library, to solve our mathematical model. The accuracy of our simulator has been validated by the Online SBML Test Suite and passed the tests for all Level 2, Version 4, and Level 3, Version 1, test cases (about 950 files).

First, we simulated our mathematical model in our simulator with a 4th order Runge-Kutta integration, but failed to produce a solution (the results of the simulations diverged) because of the stiffness of our mathematical model, which is based on the 10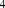 to 10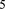 order differences of model parameters between the Hypothetical protein module, especially in the cAMP-PKA cascade, and other modules. Then we conducted the simulation again with implicit integration methods: the Adams-Moulton method and the Backward Difference (Gear's) method. Both of them solved our mathematical model appropriately and we adopted the 2nd order Adams-Moulton method for the stability of the time-evolved simulation. We set the time step for the calculation with the 2nd order Adams-Moulton method to 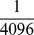 [min].

## Results

Our sustainable synapse model consists of the following five major modules; 1) Hypothetical protein module, 2) Actin control module, 3) Actin dynamics module, 4) Drebrin module and 5) Synapse dynamics module. Figure 3 shows the abstracted flow of signal transductions and the interactions of each module. A whole view of the model is shown in Figure 4, and a nested module named the 1) ‘cAMP-PKA module is involved in the 1) Hypothetical protein module. The network structure of the 1)’ cAMP-PKA module is indicated in Figure 5. The other details of each module are shown in Materials and Methods.

External stimulation, which mimics Sp-cAMPS stimuli, is positioned the most upstream in our model. The stimuli cause quantitative changes in its downstream. In the Hypothetical protein module, hypothetical proteins are induced by the stimuli. In the Actin control module, the expressions of LIMK, SSH, and COFILIN are increased. In the Actin dynamics module, the state-transition from G-actin to Dynamic F-actin is induced, and in the Drebrin module, the amount of accumulated Drebrin is increased. The details of these network connections and their dynamics will be explained in the following sections and figures. Only the Synapse dynamics module is not directly affected by the external stimuli, but is affected through the Actin dynamics module.

First, we evaluated whether our sustainable synapse model does work as an appropriate model to reproduce the experimental results. We performed simulations for the model evaluation in the following conditions.

1. The required times for gene transcription, translation and transportation to the appropriate place are all summed up as *delay* in each differential equation of protein generation.
2. We regarded the external stimuli as a rectangular pulse (Figure 6) to mimic the external stimuli procedures in Yamamoto's research [20], in which Sp-cAMPS is added to the culture medium at specific timings and completely removed after several minutes (15 min) in each experimental method. In the simulation, we added external stimuli to our model for 3 minutes with intensity: 100.

We set the length of *delay* at 60 min, based on knowledge about the necessary time for gene transcription, RNA translation and protein transportation. For the contraction of these processes to one parameter (*delay*), the question to be answered is how the length of delay affects the dynamics of the system. In the structure of the mathematical model, *delay* plays a role in suppressing the undesirable generation of synapses, which may happen with the repetitive stimuli with intervals shorter than in the conditions of the biological experiments.

We examined the effect of increasing or decreasing the value of *delay*. We tested four different conditions of *delay*; 0 (no delay), 30, 60 (two basic conditions which we can find in reference [20]) and 120 min (Figure 7). Generally, the range of the interval between stimuli, which induces long-term synaptic maintenance, became narrower with increasing *delay*. When we decreased the value of *delay*, the required range of the intervals for long-term synaptic maintenance became wider.

In detail, when we set the value of *delay* to 0 (*delay* equals 0 min), long-term synaptic maintenance was induced by three times repetition of stimuli even with no intervals. However, when the *delay* is 120 min, three times repetition of stimuli could induce synaptic maintenance only within the shorter range of intervals.

Our test indicated that a 60 min *delay* showed the best results in reproducing the experimental results [20]. It is also conceivable that a 60 min *delay* is not only suitable to fit the model with the experimental results but also suitable when we consider the time for mRNA transcription and translation, even though there may be another suitable length of delay depending on the parameters of our Hypothetical protein module. Based on the above investigation, we defined all *delays* of Proteins 1, 2 and 3 as 60 min in the following simulations.

We first evaluated the behavior of each module by comparing it with published data. After that, we tested an integrated model of all modules to see whether the model was sufficient to reproduce the sustainable synapses in the same conditions as biological experiments.

Finally, we used our model to test whether our hypothesis is critical in reproducing the sustainable synapses or not in the physiological environment.

**Integration of five different modules.** We introduced five modules and observed example dynamics of them in the model. These five modules do not work appropriately alone but interact with each other. Therefore, we have to integrate these modules to observe the whole and correct dynamics of our model. The way to integrate five modules is shown below.

- Hypothetical protein module affects Actin control module
  A hypothetical protein, Protein 3 in our model, reduces the production rate of COFILIN and SSH to repress the production of COFILIN and SSH.
- Actin control module affects Actin dynamics module
  COFILIN increases the state-transition ratio from Stable and Dynamic F-actin to G-actin and from Stable F-actin to Dynamic F-actin, because the non-phosphorylated COFILIN acts to split F-actin, and helps to bring about depolymerization from the minus end. COFILIN plays a role in promoting the state transition rate of specific transitions, from Stable F-actin to Dynamic F-actin, from Stable F-actin to G-actin, and from Dynamic F-actin to G-actin.
- The change in velocities in Dynamic F-actin and Stable F-actin reaction directly affects the production or degradation velocity of Dynamic Synapses and Stable Synapses, respectively.
- Actin dynamics module affects Drebrin module
  The Drebrin/F-actin cluster generation rate is regulated by the Stable F-actin level.
- Drebrin module affects Actin dynamics module
  The Drebrin/F-actin cluster stabilizes F-actin. So the state transition rate from Stable F-actin to Dynamic F-actin is decreased depending on the Drebrin/F-actin cluster level.

### The evaluation of the behavior of modules

We checked whether the behavior of each module of our model agrees with the published experimental data and whether the expected results produced by our model are the same as the source model. Our sustainable synapse model consists of the following modules. The Hypothetical protein module is the distinct structure of our model. We expressed our hypothesis by the sequential increase of hypothetical proteins as a response to stimuli (Figure 8). The default model involves three hypothetical proteins in this module as a three-step network succession model. In the last two sections of Results, we also used a modified model which involves only one hypothetical protein in this module. We refer to this modified model as the single step network model (Figure S1).

The cAMP-PKA module is involved in the Hypothetical protein module. We reused the model of the cAMP-PKA signaling network [52] as our cAMP-PKA module. The results of activation of PKA are shown in Figure 8.

The Actin control module in our model was built by modifying one regulation in the network of the original model [56]. We replaced the protein kinase D (PKD) in the original model with p21-activated kinase 4 (PAK4), based on published information that explains that PKD regulates COFILIN activity thorough PAK4 [62]. This knowledge is also supported by *in vivo* experiments [63]. We concluded that this published information provided sufficient evidence to change the original network component from PKD to PAK4. Our Actin control module showed an increase in the expressions of LIMK, SSH, and COFILIN as a response to Sp-cAMPS stimuli (Figure 9). This is the expected behavior of this module to transduce the signals to the next module.

The Actin dynamics module was constructed based on the description of actin dynamics in hippocampal neurons [32], [33]. The organization of Stable F-actin directly causes the increase in the number of spines [33]. Moreover this phenomenon is accompanied by L-LTP maintenance *in vivo* [32]. We confirmed that the dynamics of the F-actin to G-actin ratio during a short time (0 to 45 min) produced by our model was compatible with the results of the biological experiments ([33] Figure 3d, and Figure 11). The behavior of the three different polymerizing states of actin (G-actin (actin monomer), Stable F-actin (polymerized actin with binding proteins), Dynamic F-actin (unstable polymer of actin) represented by this module are indicated in Figure 10. About 8 days ( = 12,000 min) after three times stimuli every 12 hrs ( = 720 min, 1440 min, and 2160 min), Stable F-actin achieved a relatively higher amount among the three states of actin. This behavior of actin is reproduced in the behavior of the synapses in two different states (Dynamic synapse and Stable synapse).

A comparison of the experimental results and the results produced by our model indicates that the sum of the results of our model until the Actin dynamics module confirm the model to be successful in reproducing the dynamics of the molecules in this model.

Based on the knowledge that clustering of an actin binding protein, Drebrin may cause the further accumulation of the other functional proteins in a spine [45], we represented the simple state transition of Drebrin if this protein does exist in a monomer state or aggregated state to build a cluster by this module. The dynamics of Drebrin and the Drebrin/F-actin cluster are shown in Figure 12.

The synapse dynamics module shows the sum of the above signal transduction as the number of stable or dynamic synapses (Figure 13).

We integrated all of the above modules to build a sustainable synapse model and first verified whether the whole of the model could reproduce the experimental results in Yamamoto's work [20] (Figure 3g, Figure 5a, b in the reference).

### Time-course of relative synapse amount with single stimulation

Our sustainable synapse model includes a hypothetical network module as its key component. Next, to verify our hypothesis, we compared the following subjects between simulations and actual experimental results ([20], Figure 3g).

1. Time-course changes of relative synapse amount (%) with a single stimulation (duration of each stimulation: 3 min)
2. Time-course of relative synapse amount (%) with three times repetitive stimuli (duration of each stimulation: 3 min, interval: 1,440 min (24 hrs))
3. Synaptic maintenance response with various lengths of intervals and numbers of stimuli.
4. Synaptic maintenance response with various lengths of intervals and numbers of stimuli with the alteration in the structure of the Hypothetical protein module.

We estimated that the first three tests would reveal whether the current model structure is sufficient to represent the experimental results, and the remaining one would show whether the current model structure is required to reconstruct the physiological conditions.

**Figure 14:**
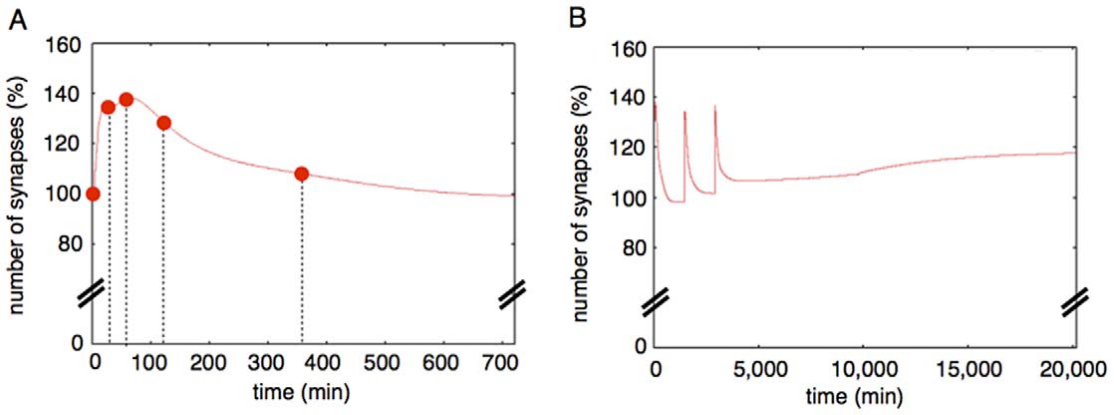
Time-course of simulation results. A: A simulation result of our mathematical model with a single stimulation. The red dots show time-points when actual experimental values are obtained [20]. The figure shows the effect of single stimuli over 12 hrs (720 min). After an additional 12 hrs (total 24 hrs = 1440 min), the effect of the single stimuli on the relative number of synapses did not change (Figure S1). B: A simulation result of our mathematical model with three times repetitive stimuli. By repeating the stimulus with a 24 hr interval ( = 1440 min) three times, the relative number of synapses was kept at a level 20% higher than the basal synapse number.

First, we simulated the time-course of the relative synapse amount (%) with a single stimulation. The duration of the single stimulation was 3 min in our condition. Based on the biological experiments, single stimulation should not be enough to induce long-term maintenance of newborn synapses [19]–[21]. We succeeded in reproducing the dynamics of synaptogenesis and maintenance which were observed by biological experiments with our model (Figure 14 **A**). A single stimulation transiently increased the number of synapses during 30 min to 2 hrs after a stimulus, but the number of synapses was decreased to basal level during the next 22 hrs in the experiments. Our simulation well reproduced the relative increases of synapses in the time-course. The graph shows the result over 12 hrs ( = 720 min), and each solid circle indicates the relative number of synapses at 30 min, 60 min, 120 min, and 360 min after the stimulus. After 12 more hours, the relative number of synapses stayed at the basal level (nearly equal to 100%); it was the same behavior as was observed with actual biological materials (figure S2).

### Time-course of relative synapse amount with three times repetitive stimuli

Next, we simulated the time-course of the relative synapse amount (%) with three times repetitive stimuli. Experiments showed that three times repetitive stimuli with appropriate intervals can maintain the synapses in the long term [19]–[21]. Our model reproduced the phenomena, excluding the exact ratio of the total number of synapses after the second and the third stimuli. Our simulation results show that 10–20% of synapses were increased in ratio with the experimental results (Figure 3.g in [20]) at those points in time (Figure 14**B**).

So far, we have confirmed that our sustainable synapse model can reproduce the long-term maintenance of synapses by three times repetitive stimuli. However, our model did not misproduce the long-term maintenance with a single stimulus.

### Synaptic maintenance response with various lengths of interval and numbers of stimuli

**Figure 15:**
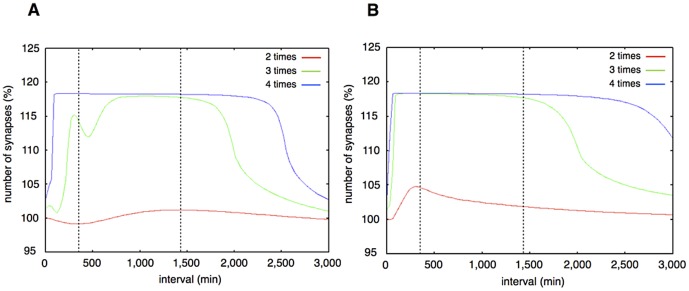
**A.** Synaptic maintenance responsiveness of our model to the number of stimuli and intervals. The horizontal axis shows the intervals of each stimulation. The vertical axis shows the increase ratio of synapses at two weeks after the first stimulation. The red line shows synaptic maintenance response with two times repetitive stimuli. The green line shows three times repetitive stimuli. The blue line shows four times repetitive stimuli. The two vertical dotted lines show the approximate lower limit and the upper limit of intervals between the three times stimuli for synaptic maintenance in experimental results [20]. **B.** Synaptic maintenance responsiveness of our model to the number of stimuli and intervals (single step model). The horizontal axis shows the intervals of each stimulation. The vertical axis shows the increase ratio synapses at two weeks after the first stimulation. Red line: two times repetitive stimuli. Green line: three times repetitive stimuli. Blue line: four times stimuli. The two vertical dotted lines show the approximate lower limit and the upper limit of intervals between three times stimuli for synaptic maintenance in experimental results [19]–[21]. This simulation is performed with the single step model. The biggest difference from Figure 15 is the upper limit of intervals which can induce synaptic maintenance.

Thirdly, to test whether our model also can reproduce the experimental results which were produced in various conditions of the interval lengths, we applied various lengths of intervals between stimuli to our model and compared the results of simulations with experimental results. We varied the range of intervals from 0 to 3,000 min (50 hrs, slightly over 2 days) for the simulation. We plotted the relative total number of synapses (the sum of the Dynamic Synapse and Stable Synapse) at 2 weeks (20, 160 minutes) after the first stimulation divided by the initial total of synapses (Figure 15**A**). Three times repetition of stimuli increased the relative total of synapses over 180 min (3 hrs) to 2,000 min (about 33 hrs). The peak value of the relative total of synapses reached about 120% (20% increase) (Figure 15**A**, Green line); that is the same increased level as in biological experiments [20]. When the stimuli were repeated twice, the increase of synapse was hardly observed (Figure 15**A**, red line). When the stimuli were repeated more than four times, the peak of the relative total of synapses was observed from 90 min to 2,500 min (about 42 hrs). The range of the peak was wider than that of the 3 times stimuli, with a similar peak value (120%) (Figure 15**A**, blue line). These simulation results agree well with the experimental results (Figure 5a, 5b in [20]).

With the three kinds of simulations above, we confirmed that more than three times repetitive stimuli with appropriate intervals are sufficient to reproduce the same phenomena as in biological experiments.

### Change in the structure of the Hypothetical protein module

The above results indicate that our sustainable synapse model is at least sufficient to reproduce the results of biological experiments.

We tested whether the structure of our sustainable synapse model is critical for the reproduction all the experimental results. To answer a part of this question, we also tried simulations with models with altered structures and tested whether these altered models also reproduced the experimental results or not.

In our model, the rate-limiting step is the Hypothetical protein module, which includes the hypothetical protein synthesis mechanism. Because the rate-limiting step dominates the whole of the reaction processes, the number of steps in the module ought to reflect the actual number of the rate-limiting reactions *in vivo*. Conversely, if the behavior of the single protein synthesis model reproduces almost the same behavior as the experimental results, the number of the newly produced proteins in the module could be one. To examine this possibility, we changed the structure of our model of the Hypothetical protein module which produces three proteins sequentially to undergo the three-step network succession, to consist of the single step network with only one hypothetical protein.

The time course of the relative number of synapses in the single step network model is shown in Figure 15**B**. This model did not explain the necessity of the time interval between stimuli. Both three times repetitive stimuli (green line) and four times repetitive stimuli (blue line) increased the relative number of synapses immediately after the external stimuli (within the first 100 min). A parameter scan was not able to solve this incompatibility between the single step network model and the experimental results. Also the upper limit of the stimulation intervals to materialize the long-term synaptic maintenance was elongated in the case of the single step network model until 3,000 min (about 75 hrs, longer than three days). This is incompatible with the experimental results (Figure 5b of [20]). The suggested upper limit of the stimulation interval with the three-step network succession model was 2,000 min (about 38 hrs). This is compatible with the experimental results, which suggested the upper limit of the effective interval between stimuli was longer than 24 hrs and shorter than 48 hrs (Figure 5b of [20]). The difference between the three-step network succession model and single step network model became more pronounced when the external stimuli were repeated more and more.

These results suggest that the Hypothetical protein module requires a multi-step network succession instead of a single step network as its mechanism to reproduce the experimental results precisely.

We additionally performed a sensitivity analysis of both the three-step network succession model and the single step network model by changing the parameter values as described in the following section.

### Effects of Parameter Changes on Sustainable Synapse Model

To evaluate the effects of the change in parameter values in the hypothetical module on the dynamics of our mathematical model, we conducted simulations in the following conditions and observed the relative number of synapses at 14 days after the first stimulation in the simulation time.

1. Change in a single parameter out of six parameters (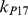, 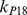, 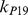, 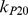, 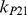, 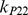) 50% above or below from the basic condition in three-step model.
2. Change in a single parameter out of six parameters (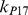, 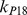, 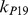, 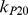, 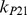, 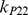) 50% above or below the basic condition in the single step model.
3. Change in a set of three parameters (set of 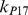, 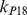, 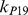 or set of 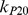, 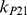, 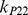) 50% above or below the basic condition in three-step model.

**Figure 16:**
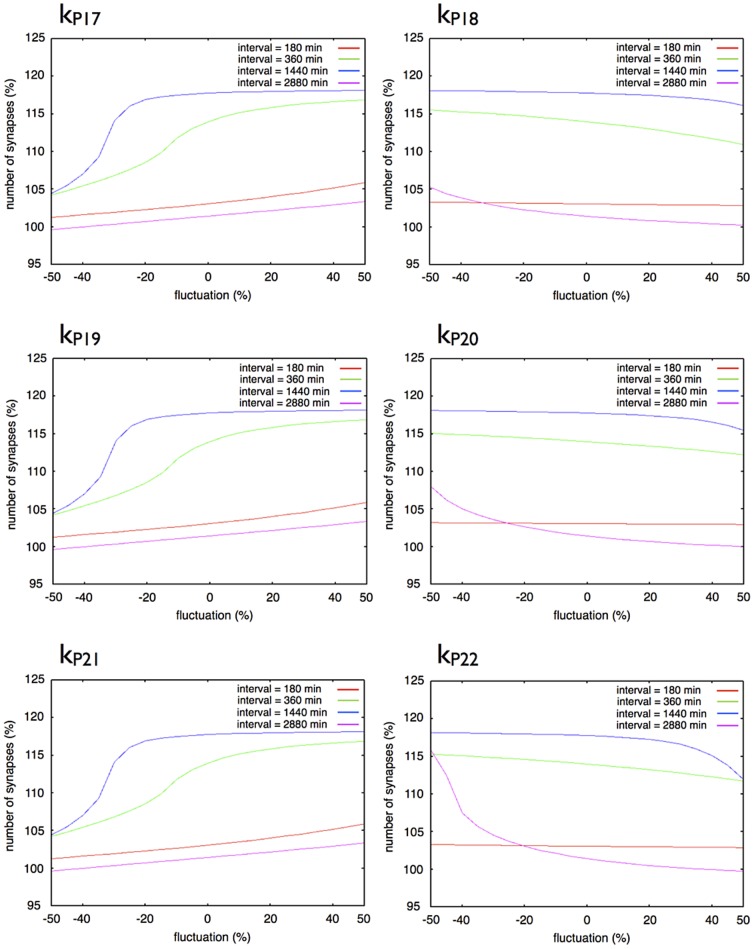
Effect of a change of a single parameter in the three-step model. Each graph shows the response of synaptic maintenance to a fluctuation in a labeled parameter. The horizontal axes in each graph show a ratio of fluctuation in a labeled parameter. The vertical axes in each graph show the relative number of synapse at 14 days after the first stimulation. The line color indicates the length of intervals between each stimulation: Red: 180 min; Green: 360 min; Blue: 1440 min; and Purple: 2880 min.

**Figure 17:**
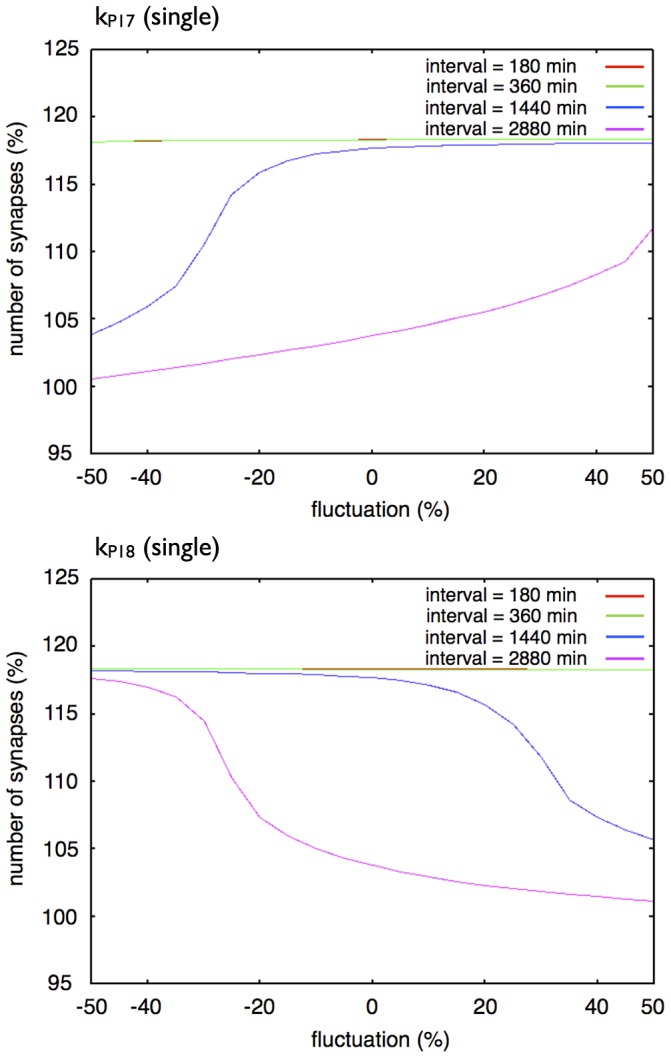
Effect of a change in a single parameter in the single step model. Each graph shows the response of synaptic maintenance to a fluctuation in a labeled parameter. The horizontal axis in each graph shows a ratio of fluctuation in a labeled parameter. The vertical axis in each graph shows the relative number of synapses at 14 days after the first stimulation. The line colors indicate the length of intervals between each stimulation: Red: 180 min; Green: 360 min; Blue: 1440 min; and Purple: 2880 min. The red line and the green line overlap throughout the entire region.

The single step network model was more sensitive to the parameter values related to protein synthesis and degradation than the three-step network succession model (Figure 16 and 17).

Figure 16 shows the changes in the dynamics of the three-step network succession model with changes in a single parameter. Almost the same trends were found when we changed 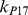, 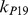 and 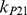, which regulate the rates of synthesis of Proteins 1, 2 and 3. In the same way when we changed 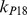, 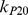 and 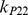 which changed the rate of degradation of Proteins 1, 2 and 3, similar trends were found. A change in 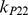 had the largest effect on the dynamics of the three-step network succession model. A change of 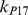, 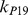 and 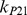 mainly affects the synaptic maintenance when intervals between stimuli are 360 min and 1,440 min, and a change in 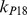, 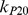 and 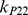 hardly affects the synaptic maintenance when intervals of stimuli are 180 min.

Figure 17 shows changes in the dynamics of our one protein synthesized model with changes in a single parameter. A change in 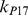 affects the synaptic maintenance when the intervals of stimuli are 1,440 min and 2,880 min, and hardly affects it when intervals of stimuli are 180 min and 360 min. A change in 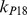 also affects the synaptic maintenance when intervals of stimuli are 1,440 min and 2,880 min, with a reverse trend when we changed 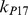, and hardly affects it when the intervals of stimuli are 180 min and 360 min. As a result, the single step model did not reproduce the experimental results [20] with adjustments in the parameters of the Hypothetical protein module.

**Figure 18:**
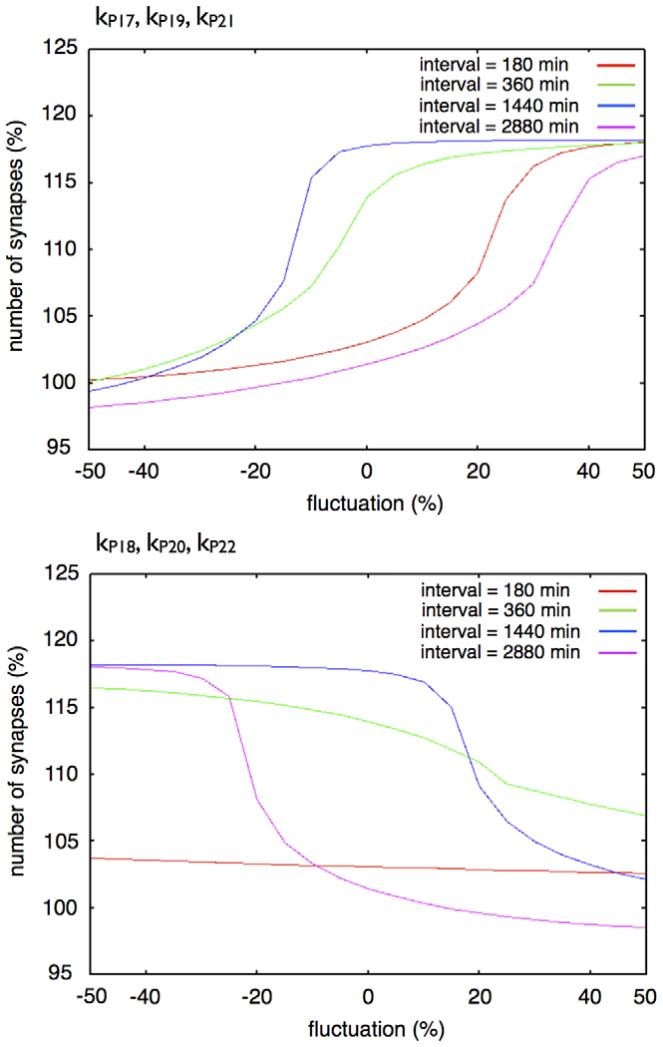
Effect of a change in a set of three parameters in the three-step model. Each graph shows the response of synaptic maintenance to a fluctuation in a set of labeled parameters. The horizontal axis in each graph shows the ratio of fluctuation of labeled parameters. The vertical axis in each graph shows the relative number of synapses at 14 days after the first stimulation. The line colors indicate the length of intervals between each stimulation: Red: 180 min; Green: 360 min; Blue: 1440 min; and Purple: 2880 min.

Figure 18 shows changes in the dynamics of our three-step network succession model with changes in a set of three parameters (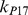, 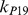, 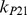 or 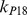, 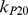, 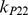). Compared with the change in a single parameter, the effects of a change in three parameter values at the same time were clearly observed. A change in 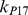, 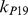, 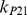 up to 20% above the basic condition and a change in 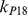, 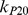, 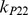 up to 20% above and below the basic condition were allowed to reproduce the experimental results.

To summarize, the three-step network succession model is more robust when subject to changes in parameter values and more suitable for reproducing the experimental results than the single step model.

## Discussion

Our model succeeded in mimicking the dynamics of synaptogenesis and synaptic maintenance with the mathematical model simulation, which occurs under cAMP and its analogue stimuli. The most characteristic feature of our model is the hypothetical signaling mechanism, which consists of the succession of the network with each stimulus. The computational simulations demonstrated that the hypothetical signaling mechanism reproduces the molecular and the cellular behaviors during synaptogenesis and their maintenance as observed in experiments, including not only the relative number of synapses produced after external stimuli, but also the duration of maintenance of these synapses, which other mathematical models had not tried to reproduce in such a long range in keeping with the experimental results [19]–[21].

The simulation results also indicate that the synaptogenesis and maintenance are induced by the repeated L-LTP dependent manner. These results mean that synaptogenesis and the maintenance require *de novo* protein synthesis and gene transcription. As is generally known, L-LTP is accompanied by protein synthesis and gene transcription. Therefore, there is no point of incompatibility between our results and the known facts. Hence, we concluded that our hypothesis is one of the conceivable mechanisms working behind the long-term maintenance of synapses.

Spike-timing-dependent plasticity (STDP) is recognized as a candidate to explain the mechanism of adjusting the strength of connections between neurons in the brain. STDP happens in a 100,000 times shorter time compared with the timescale of our model; this is 5 to 40 msec [64]. Such a phenomenon is thought to depend on the diffusion processes of small molecules. For example, the dynamics of calcium ion via the NMDA receptor is suggested as a key mechanism of STDP. The molecular mechanism of STDP suggests that STDP works in the area close to the connecting surface of the synapse. In the phenomenon represented by our model, STDP could be involved in the production of LTP as a response to each stimulus. Also the confirmed critical mechanism of STDP, that is the sequence of the spikes in pre- and postsynaptic neurons, might be essential for the basic establishment of a stable synapse.

We tested a four-step network succession model by adding a 4th hypothetical protein into our model (Figures S3 and S4). This modification of our model produced the result that the newly produced synapses by the repetitive stimuli were retained for a longer duration than that indicated by the biological experiments. This could mean that the molecular network which contributes to the memory in the experiments does not consist of the 4th succession of the network. At the same time, when a memory is retained for a longer time than we expected based on the experiments, then the molecular network could consist of more than 4 network successions. This result gave us a perspective to explain the variation of ability to memorize of individuals and generations.

NMDA receptors are thought to play an important role in producing STDP. The receptors in the synapse surface of a postsynaptic neuron receive the signal and let calcium permeate into the postsynaptic cells as it will bring about a spike. This mechanism must be required for LTP to occur with each stimulus in the upstream of the activation of PKA and the signals to activate a new protein synthesis scheme.

The working point of PKMZeta is thought to be an early step in maintaining memory [65], [66]. Previous studies on molecular networks which involve PKMZeta based on previous work suggest that if we persistently activate PKMZeta in neuronal cells, the condition could produce long-term memory by moving forward the signal to transcription factors, such as CREB. However, even if PKMZeta is active, when the protein synthesis pathway which works under PKA is disrupted, the individual can produce spines only for short time but cannot maintain them for long enough to materialize long-term memory [65] because the mechanism requires the protein synthesis under the PKA signal. This signal is positioned in the downstream of PKMZeta; this means that PKA can compensate for the function of PKMZeta in protein synthesis, but PKMZeta cannot do the inverse. This hypothesis still needs to be investigated in many aspects, for example, whether the CaM to PKA pathway is the only pathway for protein synthesis, or whether the requirement for a precise interval really produces a new protein or not.

Figure 13 shows an interesting dynamics of synaptic maintenance. The result suggests that the sustainable synaptic maintenance mechanism may have 2 states in its dynamics. At a point in time of about 8 days (around 12,000 min), the Stable Synapse showed an unsmooth curve, and increased the relative number of synapses in a switching manner from the basic state to the other. We suppose the mechanism of such behavior is mainly induced by an indirect positive feedback loop between Stable F-actin and the F-actin/Drebrin Cluster and the Hill equation [57], which we adopted as the rate equation for the F-actin/Cluster generation in the Drebrin module.

Our model seems to have an asymptotically stable state after 53,600 min in the displacement of the flux rate during a time step of total synapses (the number is smaller than 10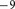 after this point in time). However we were not able to perform a precise steady state analysis based on the Eigenvalue of our 66 simultaneous equations because of the complex structure of our model. For example, there exists a free software package to analyze the steady state of a model instantly, if the model does not include a time delay [67]. However, it was not possible to solve our model with any of the free software packages currently available and it could only be solved by the original software we developed. We will develop a tool based on our original library to analyze the steady state, diverging point, etc., for further use with the models described in SBML, which include the time delay, and the other equations that cannot be solved with current ordinary equation solvers.

Our model includes hypothetical Proteins 1, 2 and 3. An important future task is the identification of the proteins which correspond with the hypothetical proteins in our mathematical model. It will help to elucidate the actual mechanism of controlling these proteins, including information about how many rate-limiting steps exist in our Hypothetical protein module or the whole system of synaptic maintenance. There exist some candidates corresponding to these proteins.

BDNF is one of the most prominent candidate proteins which seems suitable as the first transducer of the multi-step signaling. Recently, BDNF has attracted wide attention as a Plasticity Related Protein (PRP) and its functions in plasticity have been revealed [68]–[70]. It is very interesting that repeated exposure to BDNF with appropriate intervals also induces synaptogenesis and maintenance [71]. In addition, BDNF mRNA accumulates on dendritic spines when L-LTP is induced, and these accumulations require 1–2 hrs after the stimulation from the external region of a cell. This required time before mRNA accumulations seems to determine the appropriate intervals between stimulations and might be an important factor in explaining why appropriate intervals between stimuli are essential. Furthermore, BDNF signaling phosphorylates COFILIN in dendritic spines and is necessary for expansion and stabilization of dendritic spines during LTP [72], [73]. Finally, it is also suitable for our Hypothetical protein module that BDNF transcription is regulated by CREB which works in the downstream of the PKA signaling cascade [74]. All these facts are compatible with our model, hence BDNF is a suggested candidate for the concrete component of our model.

Another prominent candidate for the hypothetical protein in our mathematical model is Arc (activity-regulated cytoskeleton-associated protein), also known as activity-regulated gene 3.1 (Arg3.1). Arc mRNA traffics to dendrites and specifically accumulates at sites of synaptic activity [75]–[77]. The Arc protein also accumulates in dendrites and becomes enriched at the site of local synaptic activity, suggesting that the Arc protein is locally synthesized [78]–[80]. The Arc protein coprecipitates F-actin and relates to phosphorylation of COFILIN to stabilize skeletal F-actin, but the detailed mechanism of COFILIN phosphorylation by Arc has not been clarified. We expected the mechanism of phosphorylation of COFILIN by Arc to be that the Arc protein directly/indirectly inhibits the activity or the generation of SSH, which acts as the COFILIN phosphatase. There are some interesting and significant experimental results about Arc, in some cases with BDNF. Huang showed that local F-actin formation is necessary for selective localization of Arc mRNA to activate synapses [81]. This means that F-actin in dendritic spines helps the accumulation of Arc mRNA in activated spines. The Arc protein probably acts in the downstream of BDNF induced LTP (BDNF-LTP). BDNF-LTP induction is completely abolished by prior treatment with Arc AS [82]. The maintenance of BDNF-LTP and the associated phosphorylation of COFILIN are also rapidly reversed by Arc AS application during the critical time window [82]. Importantly, the ability of Arc AS to reverse LTP was blocked by the F-actin stabilizing drug, jasplakinolide. Together, it is reasonable to consider that the BDNF, Arc and COFILIN phosphorylation cascade which stabilizes F-actin is the essential cascade for long-term synaptic maintenance. The experimental results mentioned above do not conflict with our model description and dynamics. We are going to incorporate them in our mathematical model to make the Hypothetical protein module more concrete.

Even though we cannot prove that a three-step network succession is required, the advantages of the system compared with a single step network are clear at the points which precisely reproduce the experimental results and have a stronger tolerance to parameter changes. This evidence seems to indicate the strong possibility of the reality of a multi-step network succession. However, although our model is sufficient to represent the experimental results in the same conditions as the experiments, we admit that there may exist another system which is equally sufficient.

We simplified the involved signaling mechanisms of the Hypothetical protein module in our mathematical model in order to make clearer the point of our test. In our Hypothetical protein module, we took into account only the significance of the protein synthesis. However the transformation of the phosphorylation state of related proteins would also play important roles in synaptogenesis and maintenance through the alteration in gene expression patterns. For example, multi-step phosphorylations are often observed in signaling cascades; a good example is the MAPK pathway [28]. However, biological reactions such as phosphorylation are completed within minutes. This is a much shorter time duration than protein synthesis or gene transcription, which takes a few minutes to tens of minutes or even hours, depending on the condition of the cell.

Therefore, the difference in timescale between these biological events leads us to assume that our model is sufficient to reproduce the synaptogenesis process and maintenance, and also that our mathematical model is sufficient to suggest a new hypothesis for the mechanisms behind these events. Nevertheless it does not include the detailed components of the signaling modules. In order to analyze the acute signaling process, we may need to improve our model, including the detailed reactions which occur in a shorter time. For example, in our test for the necessary number of newly synthesized proteins, if a synthesized protein is short-lived but does not directly affect the gene expression, there could be another mechanism: synthesized proteins phosphorylate or dephosphorylate other long-lived proteins to induce synaptic maintenance. In the future, it would be meaningful to include the actual *in vivo* signaling cascades, such as various protein-protein interactions, phosphorylation and dephosphorylation.

Identification of the hypothetical proteins would also give a new perspective for the further investigation into whether there would be a mechanism controlled by a signal transduction depending on phosphorylation states of proteins or molecules included in it.

The next step for us is to perform additional experiments by varying the repeating conditions of stimulation in order to find the actual mechanism *in vivo* accurately and to enable our future work.

## Supporting Information

Figure S1 **A whole view of the single step network model.** We used a modified model from our original sustainable synapse model, which equips the three-step network succession system in the hypothetical module, which equips the single step network in the hypothetical module, shown in this figure (File: Supporting Information S1).
(PDF)

Figure S2 **Time-course of simulation results.** A simulation result during 24 hrs of the sustainable synapse model with a single stimulation. The red dots indicate the points in time when the actual experimental values are obtained [20] (File: Supporting Information 2).
(PDF)

Figure S3
**Time-course of simulation results.** A: simulation result of the 4 hypothetical protein model with a single stimulation. The red dots indicate the points in time when the actual experimental values are obtained [20]. The figure shows the effect of single stimuli over 12 hrs (720 min). B: The effect of the single stimuli on the relative number of synapses did not change until after 20,000 min ( = 14 days). C: A simulation result of the 4 hypothetical protein model with stimuli repeated three times. By repeating the stimulus with the 24 hr interval ( = 1440 min) three times, the relative number of synapses was kept at a level 20% higher than the basal synapse number after two weeks from the first stimuli. We used the same parameter values except the following parameters; 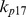 = 1.25, 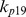 = 22.5. We added the following parameters; 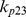 = 0.375, 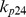 = 0.000075, when we add the equation to calculate *protein* 4 concentration (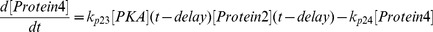). At the same time, the equation to calculate *protein* 3 concentration was redefined as follows; 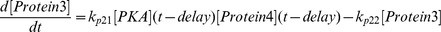.(PDF)

Figure S4 **Synaptic maintenance responsiveness of our model to the number of stimuli and intervals.** The horizontal axis shows the intervals of each stimulation. The vertical axis shows the increase in the ratio of synapses at two weeks after the first stimulation. The red line shows the synaptic maintenance response with two times repetitive stimuli. The green line shows three times repetitive stimuli. The blue line shows four times repetitive stimuli. The two vertical dotted lines show the approximate lower limit and the upper limit of intervals between three times stimuli for synaptic maintenance in experimental results [20].
(PDF)

Supporting Information S1 (XML)
