# Supplementary figures and images for: Mathematical Modeling of Sustainable Synaptogenesis by Repetitive Stimuli Suggests Signaling Mechanisms In Vivo
Source: PLoS One. 2012 Dec 20;7(12):e51000. doi: 10.1371/journal.pone.0051000 (PMC3530976; doi:10.1371/journal.pone.0051000)

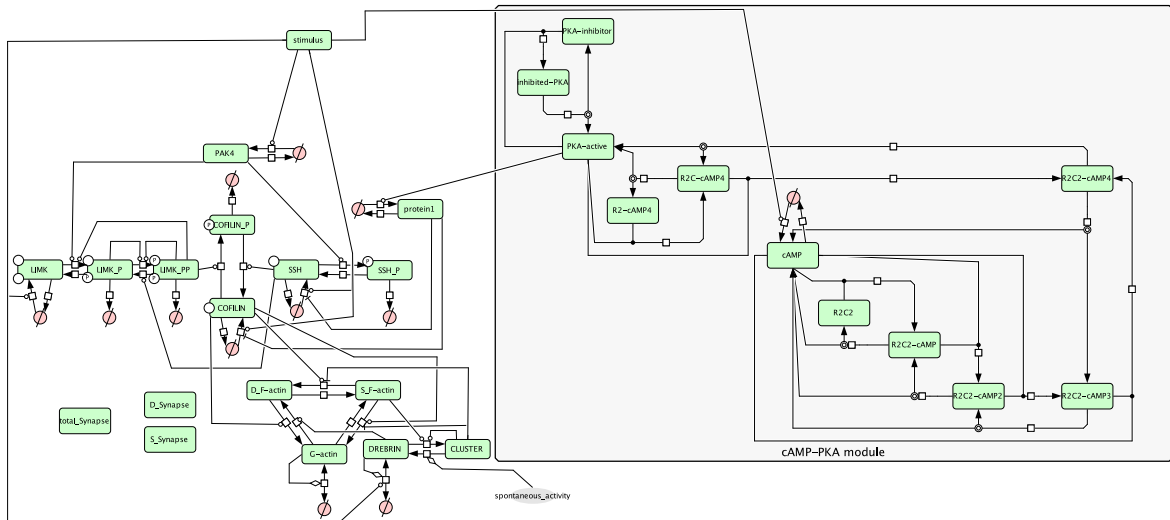

Supplement: Figure S1 — A whole view of the single step network model. We used a modified model from our original sustainable synapse model, which equips the three-step network succession system in the hypothetical module, which equips the single step network in the hypothetical module, shown in this figure (File: Supporting Information S1). (PDF) [file pone.0051000.s001.pdf]

# Supplemental Figure (additional information to Figure 14A)

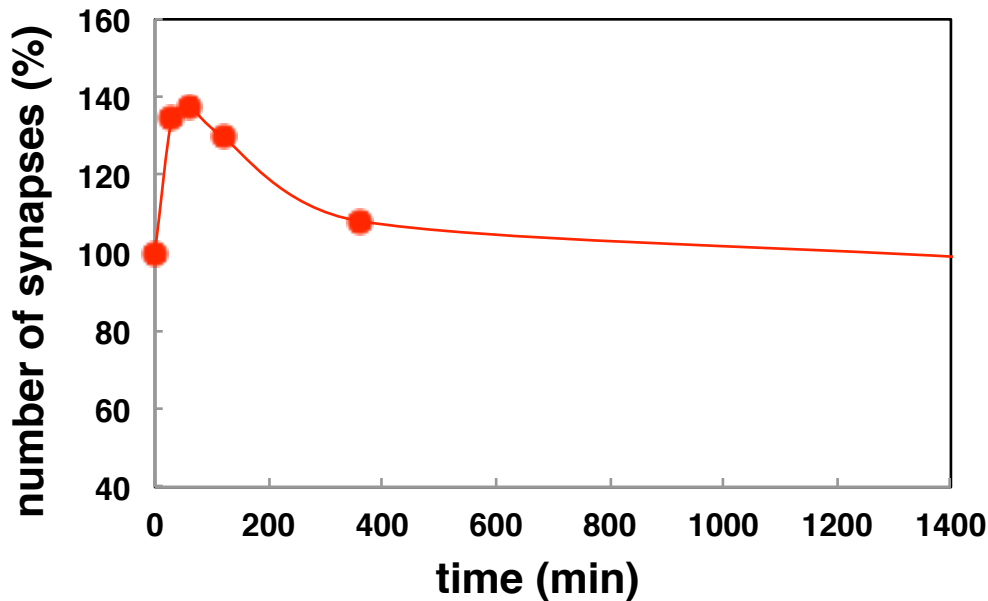

Supplement: Figure S2 — Time-course of simulation results. A simulation result during 24 hrs of the sustainable synapse model with a single stimulation. The red dots indicate the points in time when the actual experimental values are obtained [20] (File: Supporting Information 2). (PDF) [file pone.0051000.s002.pdf]

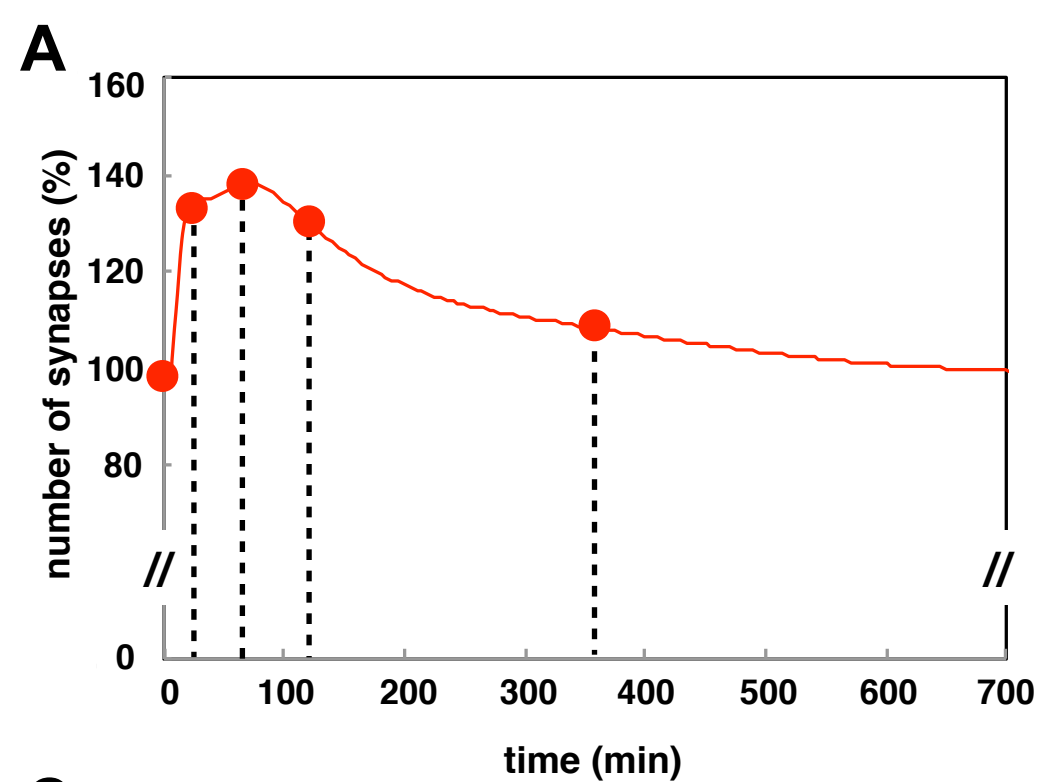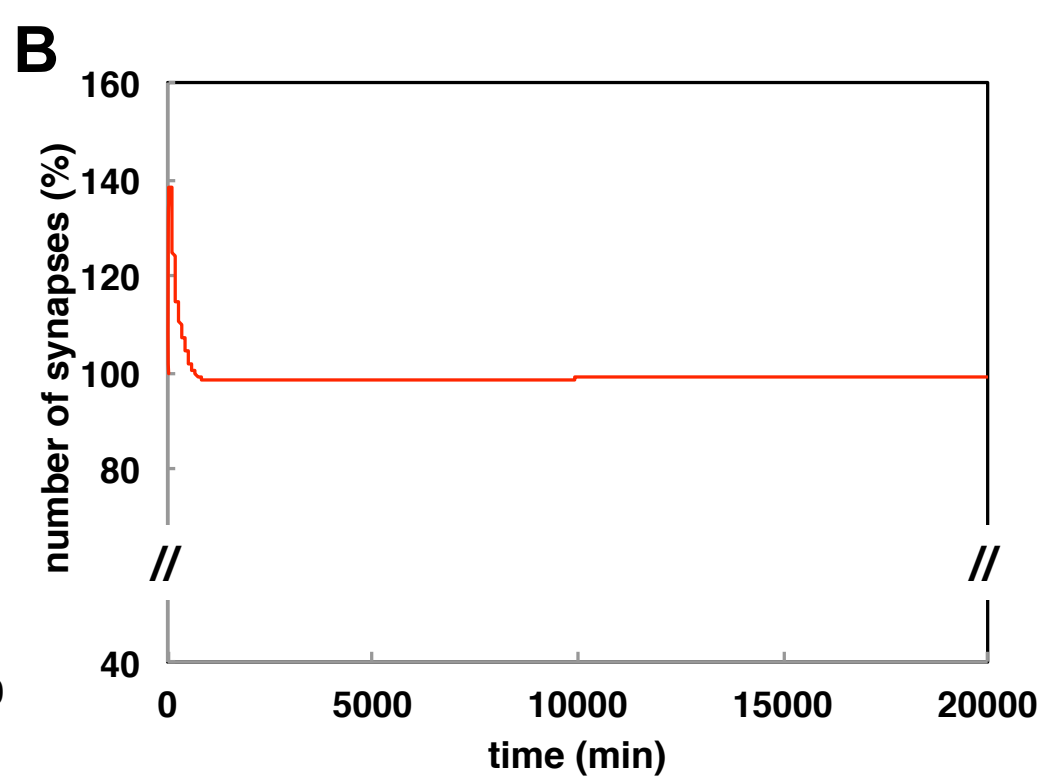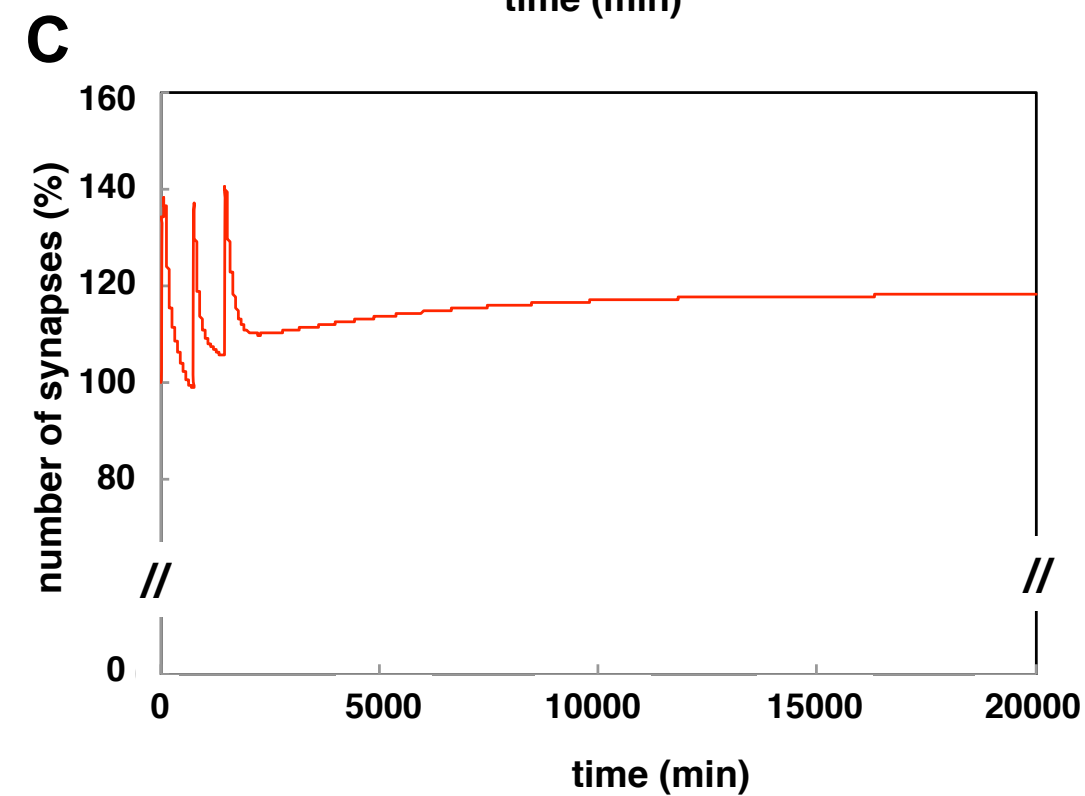

Supplement: Figure S3 — Time-course of simulation results. A: simulation result of the 4 hypothetical protein model with a single stimulation. The red dots indicate the points in time when the actual experimental values are obtained [20]. The figure shows the effect of single stimuli over 12 hrs (720 min). B: The effect of the single stimuli on the relative number of synapses did not change until after 20,000 min ( = 14 days). C: A simulation result of the 4 hypothetical protein model with stimuli repeated three times. By repeating the stimulus with the 24 hr interval ( = 1440 min) three times, the relative number of synapses was kept at a level 20% higher than the basal synapse number after two weeks from the first stimuli. We used the same parameter values except the following parameters; = 1.25, = 22.5. We added the following parameters; = 0.375, = 0.000075, when we add the equation to calculate protein 4 concentration (). At the same time, the equation to calculate protein 3 concentration was redefined as follows; . (PDF) [file pone.0051000.s003.pdf]

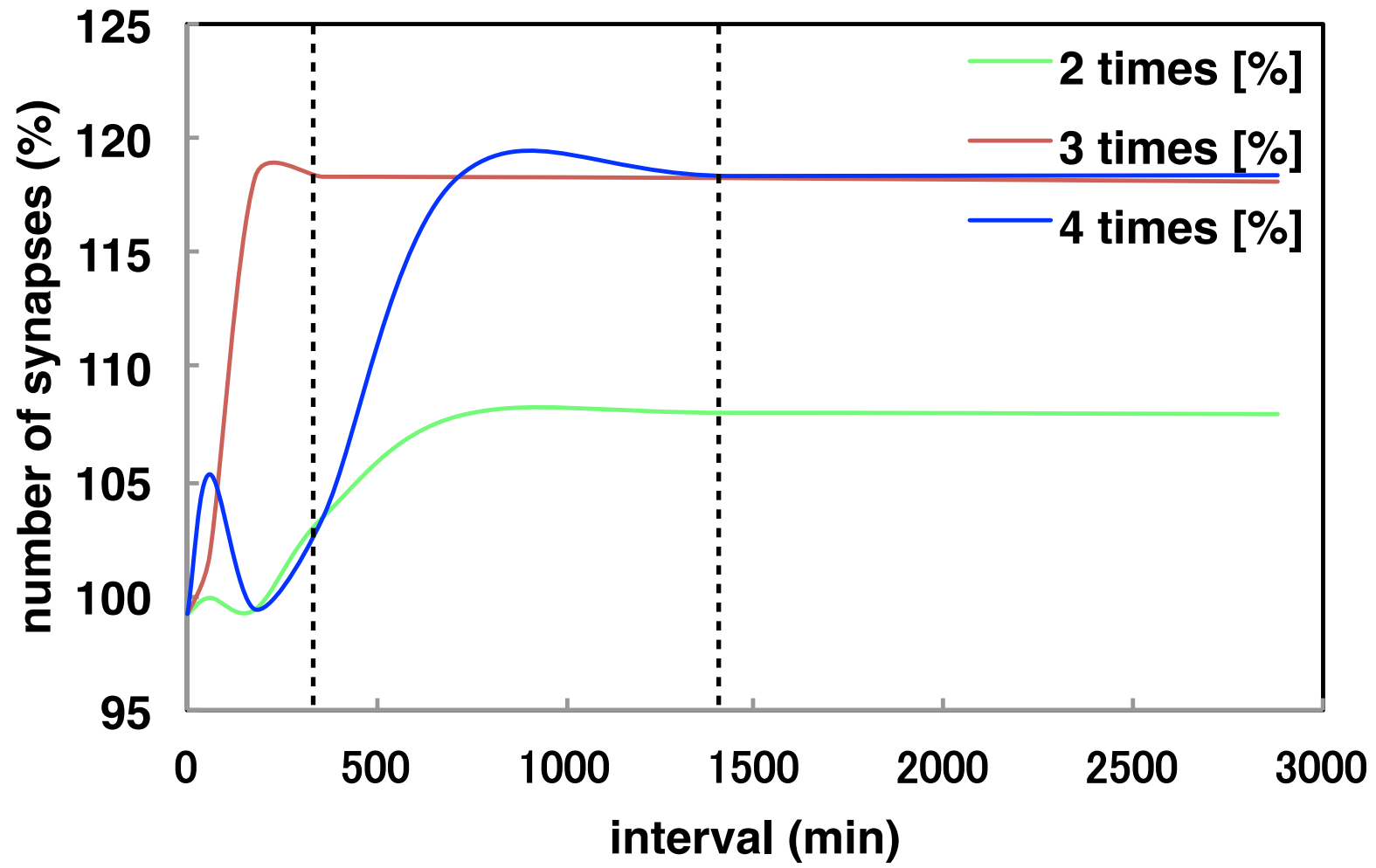

Supplement: Figure S4 — Synaptic maintenance responsiveness of our model to the number of stimuli and intervals. The horizontal axis shows the intervals of each stimulation. The vertical axis shows the increase in the ratio of synapses at two weeks after the first stimulation. The red line shows the synaptic maintenance response with two times repetitive stimuli. The green line shows three times repetitive stimuli. The blue line shows four times repetitive stimuli. The two vertical dotted lines show the approximate lower limit and the upper limit of intervals between three times stimuli for synaptic maintenance in experimental results [20]. (PDF) [file pone.0051000.s004.pdf]
